# Computational design of closely related proteins that adopt two well-defined but structurally divergent folds

**DOI:** 10.1073/pnas.1914808117

**Published:** 2020-03-18

**Authors:** Kathy Y. Wei, Danai Moschidi, Matthew J. Bick, Santrupti Nerli, Andrew C. McShan, Lauren P. Carter, Po-Ssu Huang, Daniel A. Fletcher, Nikolaos G. Sgourakis, Scott E. Boyken, David Baker

**Affiliations:** ^a^Department of Biochemistry, University of Washington, Seattle, WA 98195;; ^b^Institute for Protein Design, University of Washington, Seattle, WA 98195;; ^c^Department of Bioengineering, University of California, Berkeley, CA 94720;; ^d^Department of Chemistry and Biochemistry, University of California, Santa Cruz, CA 95064;; ^e^Department of Computer Science, University of California, Santa Cruz, CA 95064;; ^f^Department of Bioengineering, Stanford University, Stanford, CA 94305;; ^g^Joint UC Berkeley–UC San Francisco Graduate Group in Bioengineering, Berkeley, CA 94720;; ^h^Division of Biological Systems and Engineering, Lawrence Berkeley National Laboratory, Berkeley, CA 94720;; ^i^Chan Zuckerberg Biohub, San Francisco, CA 94158;; ^j^Howard Hughes Medical Institute, University of Washington, Seattle, WA 98195

**Keywords:** protein engineering, computational protein design, conformation change, calcium induced, protein switch

## Abstract

The plasticity of naturally occurring protein structures, which can change shape considerably in response to changes in environmental conditions, is critical to biological function. While computational methods have been used for de novo design of proteins that fold to a single state with a deep free-energy minimum [P.-S. Huang, S. E. Boyken, D. Baker, *Nature* 537, 320–327 (2016)], and to reengineer natural proteins to alter their dynamics [J. A. Davey, A. M. Damry, N. K. Goto, R. A. Chica, *Nat. Chem. Biol.* 13, 1280–1285 (2017)] or fold [P. A. Alexander, Y. He, Y. Chen, J. Orban, P. N. Bryan, *Proc. Natl. Acad. Sci. U.S.A.* 106, 21149–21154 (2009)], the de novo design of closely related sequences which adopt well-defined but structurally divergent structures remains an outstanding challenge. We designed closely related sequences (over 94% identity) that can adopt two very different homotrimeric helical bundle conformations—one short (∼66 Å height) and the other long (∼100 Å height)—reminiscent of the conformational transition of viral fusion proteins. Crystallographic and NMR spectroscopic characterization shows that both the short- and long-state sequences fold as designed. We sought to design bistable sequences for which both states are accessible, and obtained a single designed protein sequence that populates either the short state or the long state depending on the measurement conditions. The design of sequences which are poised to adopt two very different conformations sets the stage for creating large-scale conformational switches between structurally divergent forms.

## Significance

Computational protein design has focused primarily on the design of sequences which fold to single stable states, but in biology many proteins adopt multiple states. We used de novo protein design to generate very closely related proteins that adopt two very different states—a short state and a long state, like a viral fusion protein—and then created a single molecule that can be found in both forms. Our proteins, poised between forms, are a starting point for the design of triggered shape changes.

There are many examples of de novo designed amino acid sequences that fold to a single designed target structure (1, 2), but designing new amino acid sequences capable of adopting divergent structural conformations is challenging since the free-energy difference between the two states must be small enough that a few amino acid changes can shift the global energy minimum from one state to the other, and both states must be stable relative to the unfolded state. Redesign of natural protein backbones has yielded large conformational changes for systems, such as the Zn antenna finger, Zinc-binding/Coiled coil (ZiCo), and designed peptide Sw2, that involve changes in oligomerization state from a homotrimeric three-helix bundle to a monomeric zinc-finger fold (3–5). The pH-induced oligomerization switch (pHios) de novo design also involves a change in oligomerization state [pentamer to hexamer (6)]. In addition to changes in oligomerization state, sequence changes can introduce changes in helix orientation; in variants of the Rop-homodimer, both parallel and antiparallel helical arrangements are populated (7–9). In cases where there are not changes in oligomerization state or helix orientation, the two well-defined states are generally quite similar, for example, the dynamic switching of DANCER proteins primarily involves a single tryptophan residue (10), and the Rocker channel has two symmetrically related states that are structurally identical (11). Finally, designed protein pH- (12) and peptide- (13) dependent switches involve transitions between a single well-defined state and less well-ordered states. Overall, in most previous work, the differences in conformation have either involved changes in oligomerization state or helix orientation, been relatively small, or only one of the two states is well defined. An exception is the engineering of sequences with 98% identity that switch folds between the naturally occurring G_A_ and G_B_ domains (14).

**Fig. 1.**
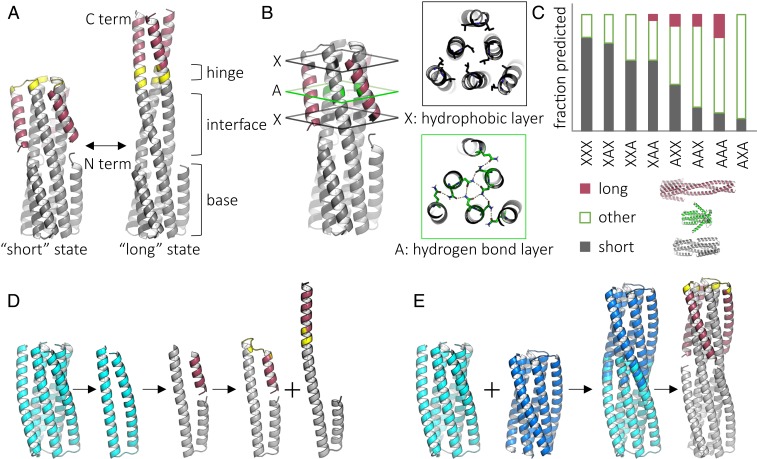
Design and in silico characterization of proteins with two distinct, well-defined ground states. (*A*) Design concept for protein backbones with a “short” and a “long” state. The backbone consists of a stable base, mobile flipping helices (red), tunable interface, and a flexible hinge (yellow). (*B*) Tunable interface between the inner helices and flipping helices. Each of three positions can be a hydrogen bond (green, “A”) or hydrophobic (black, “X”) layer. (*C*) Fraction of top 10 scored Rosetta folding predictions that resemble the short, long, or other structure for each permutation of possible interface configurations. (*D*) Initial design scheme. Starting with the previously characterized protein 2L3HC3_13 (PDB ID 5J0H) (cyan), a monomer is extracted, cut to produce a flipping helix, and reconnected with a hinge to produce the backbone of the short or long state. (*E*) Final design scheme. Previously characterized proteins 2L3HC3_13 (cyan) and 2L3HC3_23 (blue) are fused via their inner helices, outer helices are trimmed, and the hinge length is chosen such that the flipping helices pack against each other in the long state.

We sought to design sets of closely related de novo protein sequences with two structurally divergent ground states having the same oligomerization state. Inspired by class I viral fusion proteins, such as influenza hemagglutinin (15–17), we chose homotrimeric helical bundles as the fundamental architecture. We decided on a design scheme with two divergent conformations containing a constant six-helix bundle “base” and a variable portion with three inner helices and three “flipping” helices. In the compact “short” state (∼66 Å height), the flipping helices fold back to interact with the inner helices; in the extended “long” state (∼100 Å height), they extend to form interactions with each other (Fig. 1*A*). Due to the difficulty of accurately computing the relative stabilities of very different conformations, we first designed related sequences which adopt one or the other of the two states. Then, guided by experiments, we identified structural features that affect the conformational preference between states and attempted to design single sequences which adopt both states.

## Results

We started from a variant of a previously Rosetta-designed six-helix bundle [2L6HC3_13; Protein Data Bank (PDB) ID 5j0h] (18) and introduced a cut in the outer helix to produce a disconnected segment to serve as the flipping helix. This flipping helix was connected to the C terminus via either a turn to generate the short state or a contiguous helix to generate the long state (Fig. 1*D*). To determine the appropriate hinge length, connectors of two to seven residues were designed using either RosettaRemodel (19) or the insertion of short structured loops as described previously (18). Rosetta design was used to sample side-chain rotamer conformations in the context of the designed backbone, and symmetric “fold-and-dock” calculations were used to predict the three-dimensional (3D) structure based on the designed amino acid sequence (see *Materials and Methods**,* *Computational Design*). Designs were evaluated based on how well the predicted structure matched the design model (*SI Appendix*, Fig. S1); for example, in the long state, we found that a helical hinge connector of five residues resulted in the lowest-energy solution with the best packing between helices (*SI Appendix*, Fig. S1*B*).

We selected sequences that were strongly predicted to fold into one of the two states (*SI Appendix*, Fig. S1*D*), genes encoding such designs were obtained, and the proteins were purified and characterized. All initial designs were solubly expressed and had circular dichroism (CD) spectra indicative of well-folded, alpha-helical structures; three of four were trimeric by size exclusion chromatography with multiangle light scattering (SEC-MALS) (*SI Appendix*, Fig. S2). However, by small-angle X-ray scattering (SAXS), it was difficult to determine which state (short, long, or a combination) the proteins adopted (*SI Appendix*, Fig. S2).

To facilitate discrimination between the two states by SAXS, we carried out a second round of design with 1) longer flipping helices (which resulted in a greater difference in length between the states and hence more differently predicted solution-scattering profiles) and 2) helical interfaces redesigned to favor the short state (with the sequence optimized for the turn and packing of the flipping helices on the inner helices) or the long state (with the sequence optimized for packing between the flipping helices across the symmetric interface). The previous design round had enforced the same sequence at the interface regions for all designs (*SI Appendix*, Fig. S3). The length of the flipping helix was increased from 15 to 21 residues, and the hinge length changed to three residues. The proteins in this second round of designs expressed solubly, and three of the four were helical by CD spectroscopy and ran as trimers by SEC-MALS (*SI Appendix*, Fig. S4). However, by SAXS all three appeared to be in the long state (*SI Appendix*, Fig. S4), suggesting that the short state in this design scheme has higher free energy than the long state.

**Fig. 2.**
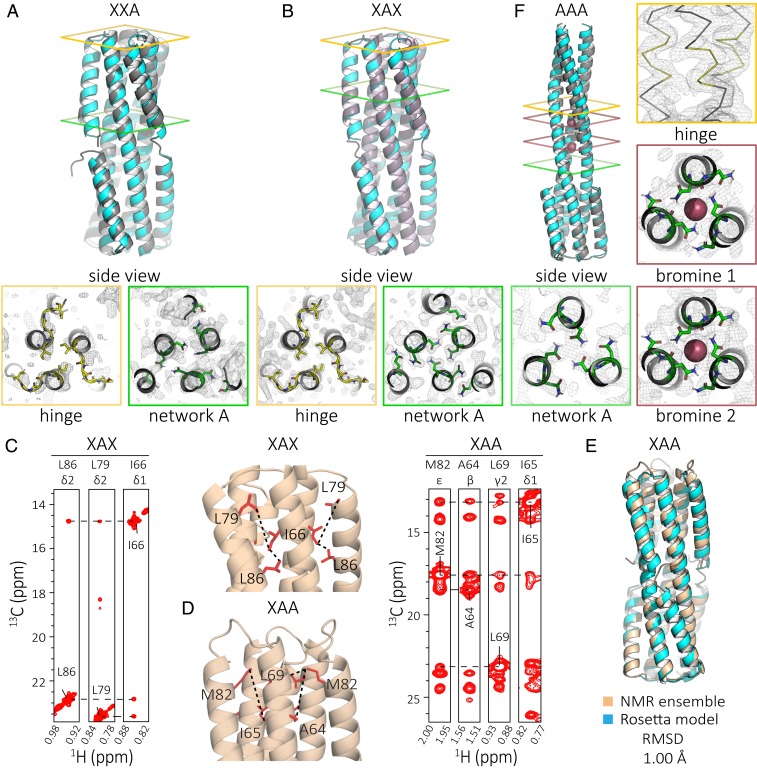
Modifying the number and position of hydrogen bond networks in the interface region changes the conformational state. (*A*) Crystal structure (gray) compared to model (cyan) of XXA (PDB ID 6NYI) shows the protein in the short state (rmsd = 1.37 Å). (*B*) Crystal structure (gray) compared to the Rosetta model (cyan) of XAX (PDB ID 6NYE) shows the protein in the short state (rmsd = 1.40 Å). (*C*) 3D C_M_-C_M_H_M_ NOESY strip for XAX is consistent with the short conformation of the protein. (*D*) 3D C_M_-C_M_H_M_ NOESY strip for XAA is consistent with the short conformation of the protein. (*E*) De novo NMR structure (PDB ID 6O0I) calculated using chemical shifts, long-range NOEs, and RDC data (beige) compared to the Rosetta model (cyan) shows the closed conformation (rmsd = 1.00 Å). (*F*) Crystal structure (gray) compared to the model (cyan) of AAA (PDB ID 6NX2) shows the protein in the long state with ion (red spheres) coordination sites (rmsd = 1.40 Å).

Our third round of designs aimed to generate short and long states with more equal stability. We used a larger portion of 2L6HC3_13 as the base to provide more stability, and fused it with a second Rosetta-designed six-helix bundle (2L6HC3_23) (18) by aligning the matching superhelical parameters of the inner three helices (Fig. 1*E*). Compared with the second-generation designs, in the short state the flipping helix was positioned closer to the inner helices and the termini of the hinge region were closer together, allowing closer packing in the short state (*SI Appendix*, Fig. S5). Four designs with different fusion sites, placement of hydrogen bond networks, and loop flexibilities were tested; all expressed solubly and were helical by CD, but the two larger designs were not trimeric by SEC-MALS (*SI Appendix*, Fig. S6). One design was confirmed to be in the long state by crystallography, again indicating that the short state had higher energy than the long state (Fig. 2*F*).

### Tuning the Short-/Long-State Difference with Hydrogen Bond Networks.

To tune the relative stability of the short and long states, we introduced modular hydrogen bond networks buried in the short state, but partially exposed in the long state. The interface strength between the inner and flipping helices is expected to depend on both the number and position of the hydrogen bond networks [replacing hydrophobic packing with more polar networks results in weaker interfaces (18), and solvent exposed locations are more likely to be disrupted by competing water interactions]. This strategy had the added advantage of creating more soluble proteins because residues necessarily exposed in the long state—when the flipping helix extends, parts of the inner helices it previously packed against are solvent exposed—are not entirely hydrophobic (as they would be if the stabilizing interactions were entirely nonpolar, as in most design calculations). The interaction energy between the flipping helix and the inner helices in the short state was tuned by combining hydrogen bond layers (“A”) or hydrophobic layers (“X”) in different orders in each of three possible positions (18) (Fig. 1*B*). Designs were given three-letter names corresponding to the layers in order, starting at the hinge proximal position. Structure prediction calculations (Rosetta symmetric fold-and-dock) suggested that some layer combinations (such as XAX) would be more favorable for the short state than others (such as AAA) and that the short and long backbones states would be among the lowest-energy states of these systems (Fig. 1*C*).

We constructed all eight possible permutations of two different layers in three positions, dubbed SERPNTs (in silico engineered rearrangeable protein topologies). We set the three hinge residues to glycine to reduce favorable contributions to the long-state free energy from the helical form of the hinge. All eight constructs expressed well, and by SEC most samples were monodisperse or had a small aggregation peak, with only AXA forming a large soluble aggregate (*SI Appendix*, Fig. S7*C*). SEC-MALS measurements indicated that, in solution, XAX, XAA, and AAA were trimeric as expected; CD wavelength and temperature measurements for XAX, XAA, and AAA showed that the designs were helical and highly temperature stable (*SI Appendix*, Fig. S7 *D* and *E*).

The number and position of the hydrogen bond networks appears to determine the state which is populated—designs with one network folding into the short state, with two networks, into a more dynamic short state, and with three networks, into the long state (see details in *SI Appendix*). Crystal structures of designs XXA (PDB ID 6NYI) and XAX (PDB ID 6NYE) matched the modeled short state (Fig. 2 *A* and *B*). For both, most of the deviation, as expected, was in the flexible hinge: the crystal structures showed that each monomer adopted a different loop. The hydrogen-bond network residues were closer to the computational model in XAX than in XXA, likely because they were buried in the former and solvent exposed in the latter. By NMR, the average solution structure adopted by XAX was locked in the compact state, as shown by the presence of multiple NOEs from a 3D C_M_-C_M_H_M_ nuclear Overhauser enhancement spectroscopy (NOESY) spectrum (Fig. 2*C*). Analysis of an MAI(LV)^proS^ methyl-labeled XAA sample also suggested a short state, with the flipping helix shifted up (toward the hinge) compared with the design model (Fig. 2 *D* and *E* and *SI Appendix*, Fig. S8). The flipping helix of XAA appears to have a more flexible interface (relative to XAX), as evidenced by increased linewidths in both the amide transverse relaxation optimized spectroscopy (TROSY) and methyl heteronuclear multiple quantum coherence (HMQC) spectra (*SI Appendix*, Fig. S9, *Left*). The crystal structure of design AAA (PDB ID 6NX2) was in the long state with some deviation from the predicted model because the asparagines of two of the hydrogen bond networks coordinated bromide ions and shifted the helical register (ions and waters were not explicitly accounted for during modeling) (Fig. 2*F* and *SI Appendix*, Fig. S10). These results suggest the potential for a single sequence that can interconvert between the two designed states since a change in three residues, corresponding to the location of a hydrogen bond network, can switch the observed state (i.e., XAA vs. AAA).

### Tuning the Short-/Long-State Free-Energy Difference in the Hinge Region.

**Fig. 3.**
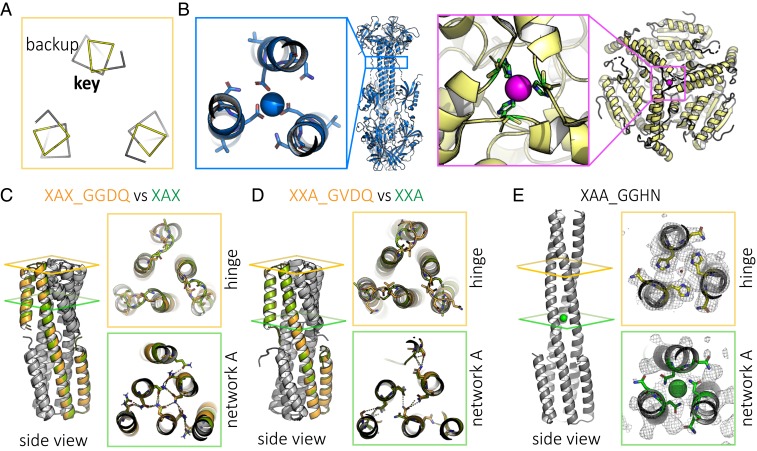
Tuning the hinge region can change the protein state. (*A*) Positions of key hinge residue G75 and backup hinge residue T76 have the potential to form an ion coordination site in the long state, shown here, but not in the short state. (*B*) Examples of natural sites include calcium-coordinating residues of human cytomegalovirus glycoprotein B (PDB ID 5CXF; blue) and nickel-coordinating residues of methylmalonyl CoA decarboxylase (PDB ID 1EF8; yellow). (*C*) Crystal structure of hinge modification XAX_GGDQ (orange; PDB ID 6NYK), like its parent XAX (green), is in the short state. (*D*) Crystal structure of hinge modification XXA_GVDQ (orange; PDB ID 6NZ1), like its parent XXA (green), is in the short state. (*E*) Crystal structure of hinge modification XAA_GGHN (PDB ID 6NZ3), unlike parent XAA, is in the long state.

To develop amino acid sequences for which the free energies of the short and long states would be still closer together, we next tuned the hinge region, which transitioned from loop to helix in the conformational change (Fig. 2 *A*, *B*, and *E* vs. Fig. 2*F*). In the long state but not in the short state, the “key” hinge residue (G75) and the “backup” hinge residue (T76) have the potential to form an ion coordination site (Fig. 3*A*). Similar natural sites include the calcium coordination site in human cytomegalovirus glycoprotein B (PDB ID 5CXF) (20) and the nickel coordination site in methylmalonyl CoA decarboxylase (PDB ID 1EF8) (21) (Fig. 3*B*), among others (*SI Appendix*, Figs. S11–S13) (22). We sought to design calcium- or nickel-binding sites in the long state by introducing aspartic acid or histidine at the key hinge residue position. We also designed for structural ambiguity in the turn—with the result that the secondary structure prediction for residue 75 is mixed helical and coil, but with low confidence (*SI Appendix*, Fig. S14).

Crystal structures showed that designs XAX_GGDQ (PDB ID 6NYK) and XXA_GVDQ (PDB ID 6NZ1), which introduced a potential calcium coordination site, adopted the short state like their respective parent designs without the hinge residue modifications (i.e., XAX and XXA) (Fig. 3 *C* and *D*). In contrast, the crystal structure of XAA_GGHN (PDB ID 6NZ3), which introduced a potential nickel coordination site (occupied by a water molecule in the structure), was in the extended state, unlike the parent XAA design, with rmsd 1.44 Å to the extended model (Fig. 3*E*). Together, these results show that the hinge region is amenable to mutation without causing the protein to misfold into undesigned conformations.

### A Bistable Designed Sequence That Adopts Both Short and Long States.

Having found that both the short- and long-state backbones are compatible with modular replacement of hydrogen-bonding layers between the helices and with mutations in the hinge region, we carried out more detailed structural characterization to determine if any of the designs could adopt both conformations. Crystal structures were determined for all potential bistable candidates (XAX_GGDQ, XXA_GVDQ, XAA_GGHN, and XAA_GVDQ), and we chose to focus on XAA_GVDQ because of the presence of a bound calcium. Detailed NMR and crystallographic characterizations of design XAA_GVDQ, described in the following paragraphs, suggest that this sequence may indeed populate both short and long states.

**Fig. 4.**
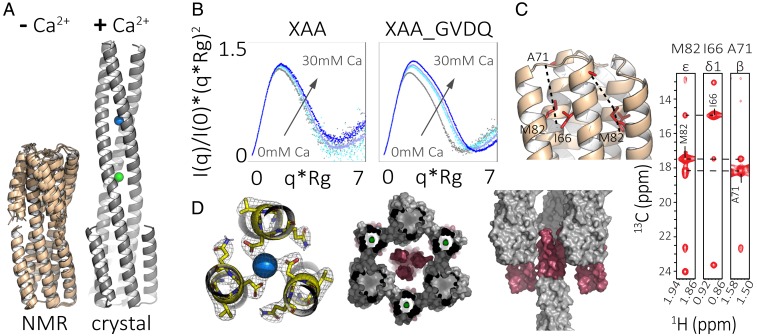
Bistable design XAA_GVDQ is in the short state by NMR and in the long state by crystallography. (*A*) Summary of crystallographic and NMR characterization of XAA_GVDQ with and without calcium: (*Left*) Rosetta models based on RDC and chemical shift data show the protein to be in the short state without calcium (PDB ID 6O0C); (*Right*) crystal structure shows the protein to be in the long state with calcium (blue) and chloride (green; PDB ID 6NY8). (*B*) Normalized Kratky plots from SAXS experiments for control protein XAA (linker GGGT) and sample protein XAA_GVDQ measured with up to 30-mM calcium. (*C*) 3D C_M_-C_M_H_M_ NOESY strips of XAA_GVDQ indicate the protein as in the short state. (*D*) Crystallographic results at the hinge and hydrogen bond networks with calcium shown as a blue sphere (*Left*); crystal packing from two different views with the flipping helix highlighted in red (*Middle* and *Right*).

To characterize the structure in solution, we prepared a selective MAI(LV)^proS^ methyl-labeled XAA_GVDQ sample (*SI Appendix*, Fig. S9, *Right*). In the absence of calcium, the 3D CM-C_M_H_M_ NOESY and residual dipolar couplings (RDCs) were consistent with the short state. We solved the NMR structure using the chemical shifts, long-range NOEs (both methyl to methyl and amide to methyl), and RDCs, and found it to be very close to the short-state design model with an rmsd of 1.19 Å (Fig. 4 *A* and *C* and *SI Appendix*, Fig. S15 and Table S6; PDB ID 6O0C).

We next sought to determine the structure of the design in the presence of calcium. Comparison of RDCs, the two-dimensional (2D) ^1^H-^15^N amide TROSY spectrum, and 2D ^1^H-^13^C methyl HMQC recorded using XAA_GVDQ samples with and without calcium (*SI Appendix*, Fig. S15) showed that the average solution structure in the presence of calcium remains in the short state and exhibits a range of nonspecific chemical shift perturbations at solvent-exposed residues. Overlays of one-dimensional (1D) methyl proton spectra showed a significant loss of signal in a calcium-concentration–dependent manner due to the formation of a nonobservable state, likely a higher-order oligomer or aggregate (*SI Appendix*, Fig. S15). SAXS analysis of XAA_GVDQ and the control sequence with no aspartate, XAA (linker GGGT), showed calcium-dependent changes in the normalized Kratky plot, indicating an increase in size (Fig. 4*B* and *SI Appendix*, Table S4).

Because calcium-induced oligomerization or aggregation complicated NMR structure determination of the calcium-bound state, despite efforts to optimize pH, temperature, concentration, calcium, and buffer components, we turned to X-ray crystallography and sought to determine crystal structures in both the absence and presence of calcium. After extensive screening, we were able to solve a structure in the absence of calcium (*SI Appendix*, Fig. S17, PDB ID 6NXM) in which the flipping helices interacted with the inner helices in the same manner as they would in the designed short state, but did so in a domain-swapped manner with a symmetry mate to form a hexamer. This was consistent with the observation of the short state by NMR in the absence of calcium; the domain swapping almost certainly arose due to crystallization conditions.

We next sought to solve the structure of the design in the presence of calcium. We succeeded in solving the structure in 50-mM calcium acetate by molecular replacement to 2.3-Å resolution (*SI Appendix*, Table S3). The protein was in the designed long state with a metal ion coordinated by the aspartates in the hinge region (PDB ID 6NY8; Fig. 4 *A* and *D*). The ion was most likely calcium given the coordination and crystallography conditions, which consisted of 0.05-M calcium acetate, 0.1-M sodium acetate pH 4.5, 40% (wt/vol), and 1,2-propanediol at 18 °C.

It is plausible that a calcium-bound long state similar to that observed in the calcium-bound crystal structure was transiently populated in solution in the presence of calcium, along with the short apo-state. This state may have been more prone to aggregation, as suggested by the calcium-dependent signal loss in the NMR experiment, which was carried out at high protein concentrations. Alternatively, it is possible that the long state in the crystal structure was not populated in solution conditions and was instead driven by crystallization conditions and forces rather than ion coordination, but these crystal-specific interactions would likely have been quite small as the flipping helix made relatively few crystal-lattice interactions (Fig. 4*D*).

## Discussion

We designed a set of de novo proteins in which small changes (three mutations) in the interface or hinge region cause the proteins to fold into two distinct and structurally divergent conformations. In contrast to most previous work, the differences in conformation of our designs are relatively large, they do not rely on changes in oligomerization state, and both states are well defined. Most notably, the protein XAA_GVDQ adopts the designed short state by NMR in the absence of calcium, and the designed long state by crystallography in the presence of calcium. The extensive tuning needed to find conditions in which our designed proteins adopt two states, and the challenge posed by the aggregation tendency of one of the states, highlights the remarkable properties of natural two-state proteins (such as viral fusion proteins) that convert between two well-behaved but very different conformations.

Given that changes in three residues in the hinge region can change the conformation of the design (hinge GGHN is long; GGGT is short), future work on these residues may yield designs that populate soluble short and long states in equilibrium and are switchable in solution conditions. Our designed systems, poised as they are between two different states, are excellent starting points for design of triggered conformational changes—only small binding free energies will be required to switch conformational states; a particularly interesting possibility is synthetic environmentally sensitive membrane fusion proteins analogous to those of the viral proteins that inspired the design scheme.

## Materials and Methods

### Computational Design.

To connect the backbone after the outer helix cut was introduced (Fig. 1 and *SI Appendix*, Fig. S1), RosettaRemodel was used to insert helical fragments for the long state; a blueprint file was used to specify the length (2–7) and secondary structure (i.e., H) of the insert (19). For the short state, a database lookup method was used to insert small, structured loops (helix-turn-helix motifs) as described previously (18, 23).

Amino acid sequence and side-chain rotamer conformations of the flipping helices, hinge connectors, and surrounding regions were optimized using RosettaDesign (24) calculations carried out as described previously (18). Early designs also utilized Rosetta Multistate (25) to identify amino acid sequences that resulted in favorable side-chain packing in both short and long states (*SI Appendix*, Fig. S1 *C* and *D*). Hydrogen bond networks were designed and evaluated using the HBNet (18) method in Rosetta (26). Hydrogen bond network residues were constrained during design using AtomPair constraints on the donors and acceptors of the hydrogen bonds as described previously (18).

Amino acid sequences of the short, structured loops were chosen based on how closely the torsion angle preference of a given sequence [ABEGO torsion bin, where “A” indicates the alpha region of the Ramachandran plot; “B,” the beta region; “E” and “G,” the positive phi region for beta strands and helices respectively; and “O,” the cis peptide conformation (27)] matched the torsion angles of the inserted loop backbone (28); sequences that have a high propensity to adopt the structure of the designed helix-turn-helix backbone should favor the short state, whereas sequences that deviate from ideal torsional preferences or contain flexible residues, such as glycine, should allow for the flipping helix to deviate from the short state.

To redesign hydrogen bond layers to hydrophobic layers and minimize surface-exposed hydrophobics, and to reinforce packing around new loop sequences, designable positions were designated in a resfile and side chains were sampled without backbone movement using PackRotamersMover in RosettaScripts (29). Backbone movement was disallowed to minimize disruption of the hydrogen bond network.

To predict whether a given designed sequence would prefer the short backbone, extended long backbone, or a mix of both, symmetric fold-and-dock calculations were performed (30); results were evaluated by symmetric rms distance to measure how closely the structures predicted to be the lowest-energy matched each of the short and long designed backbones (Fig. 1*C* and *SI Appendix*, Fig. S1*D*). Designs and fold-and-dock outputs were also manually inspected to identify surface-exposed hydrophobic residues, which could lead to aggregation problems once the proteins were purified. Energy calculations used the then default Rosetta score function talaris2013 (31) for fold-and-dock calculation and the then experimental ref2015 (32) for design calculations.

Note that all calculations were done using symmetry flags or the symmetric versions of protocols to specify the three-fold rotational symmetry (C3) of the molecules.

Online RosettaCommons documentation links are as follows: RosettaRemodel, https://www.rosettacommons.org/docs/latest/application_documentation/design/rosettaremodel; Multistate Design, https://www.rosettacommons.org/docs/latest/application_documentation/design/mpi-msd; HBNet, https://www.rosettacommons.org/docs/latest/scripting_documentation/RosettaScripts/Movers/movers_pages/HBNetMover; Constraint File, https://www.rosettacommons.org/docs/latest/rosetta_basics/file_types/constraint-file; Resfile, https://www.rosettacommons.org/docs/latest/rosetta_basics/file_types/resfiles; PackRotamersMover, https://www.rosettacommons.org/docs/latest/scripting_documentation/RosettaScripts/Movers/movers_pages/PackRotamersMover; RosettaScripts, https://www.rosettacommons.org/docs/latest/scripting_documentation/RosettaScripts/RosettaScripts; Fold and dock, https://www.rosettacommons.org/docs/latest/application_documentation/structure_prediction/fold-and-dock; Score Functions, https://www.rosettacommons.org/docs/latest/rosetta_basics/scoring/score-types; Symmetry, https://www.rosettacommons.org/demos/latest/tutorials/Symmetry/Symmetry.

### Synthetic Gene Construction.

Synthetic genes were ordered from Genscript Inc. and delivered in pET28b+ *Escherichia coli* expression vectors, inserted at the NdeI and XhoI sites. Genes were encoded in frame with the N-terminal hexahistidine tag and thrombin cleavage site. A stop codon was added at the C terminus just before XhoI.

### Bacterial Protein Expression and Purification.

Plasmids were transformed into chemically competent *E. coli* BL21 Star (DE3) (Invitrogen) or Rosetta (DE3)pLysS (QB3 MacroLabs). Starter cultures were grown in lysogeny broth (LB) media with 50-μg/mL kanamycin overnight with shaking at 37 °C. Five milliliters of starter culture was used to inoculate 500-mL 2xTY with 50- to 100-μg/mL kanamycin at 37 °C until optical density (OD) 600 = 0.6. Cultures were cooled to 18 °C and induced with 0.1-mM isopropyl β- d-1-thiogalactopyranoside (IPTG) at OD 600 = 0.8 and grown overnight. Cells were harvested by centrifugation at 3,500 rcf for 15 min at 4 °C and resuspended in lysis buffer (25-mM Tris pH 8.0, 300-mM NaCl, 20-mM imidazole), then lysed by sonication. Lysates were cleared by centrifugation at 14,500 rcf for at least 45 min at 4 °C. Supernatant was applied to 0.5- to 1-mL Ni-NTA resin (Qiagen) in gravity columns preequilibrated in lysis buffer. The column was washed thrice with 10 column volumes (CVs) of wash buffer (25-mM Tris pH 8.0, 300-mM NaCl, 20-mM imidazole). Protein was eluted with 25-mM Tris pH 8.0, 300-mM NaCl, 250-mM imidazole, and 0.5-mM tris(2-carboxyethyl)phosphine (TCEP). Proteins were buffer exchanged using Akta Pure FPLC (GE Healthcare) and a HiPrep 26/10 desalting column (GE Healthcare) into 25-mM Tris pH 8.0, 300-mM NaCl. The hexahistidine tag was removed by thrombin cleavage by incubating with 1:5,000 thrombin (EMD, Millipore) overnight at room temperature. Proteins were further purified by gel filtration using FPLC and a Superdex 75 10/300 GL size exclusion column (GE Healthcare).

### CD Measurements.

CD wavelength scans (260 to 195 nm) and temperature melts (25 to 95 °C) were measured using an AVIV model 410 CD spectrometer. Temperature melts monitored absorption signal at 222 nm and were carried out at a heating rate of 4 °C/min. Protein samples were prepared at 0.25 mg/mL in PBS in a 1-mm cuvette.

### SAXS Measurements.

Proteins were purified by gel filtration using Akta Pure FPLC and the Superdex 75 10/300 GL into SAXS buffer (20-mM Tris pH 8.0, 150-mM NaCl, and 2% glycerol). Fractions containing trimers were collected and concentrated using Spin-X UF 500 10 molecular weight cut-off (MWCO) spin columns (Corning), and the flow-through from the spin columns was used as buffer blanks. Samples were prepared at concentrations of 3- to 8-mg/mL. SAXS measurements were made at the SIBYLS 12.3.1 beamline at the Advanced Light Source (ALS). The light path was generated by a superbend magnet to provide a 1,012-photon/s flux (1-Å wavelength) and detected on a Pilatus3 2M pixel array detector. Each sample was collected multiple times with the same exposure length, generally every 0.3 s for a total of 10 s, resulting in 30 frames per sample.

Each dataset plotted was a separately experimentally measured sample and initially processed in ScÅtter (https://bl1231.als.lbl.gov/scatter/). The normalized Kratky plots were structurally interpreted by evaluating the location of the peak relative to a reference sample. SAXS profiles were also analyzed using the FoXS Server (https://modbase.compbio.ucsf.edu/foxs/) (33, 34). For the best analysis consistency, we examined the fit of the data to each of the two states separately (rather than as an ensemble of both states using the FoXS server by providing both models or using the MultiFoXS protocol) and made the simplifying assumption that the better fit represented the dominant state in solution.

### Protein Crystallography, X-Ray Data Collection, and Structure Determination.

Proteins were purified by gel filtration using Akta Pure FPLC and the Superdex 75 10/300 GL into 20-mM Tris pH 8.0, 150-mM NaCl and concentrated to 3 to 10 mg/mL. Most commonly, 8-mg/mL samples were screened using a Mosquito (TTP Labtech) with JCSG I-IV (Qiagen), Index (Hampton Research), and Morpheus (Molecular Dimensions) screens. Sitting drops of 1:1 mixture of 150- to 200-nL protein and 150- to 200-nL reservoir solution were incubated at 4 °C or 18 °C. Crystallization conditions are provided in *SI Appendix*, Table S2. The X-ray datasets were collected at the Berkeley Center for Structural Biology beamlines 8.2.1 and 8.3.1 of the ALS at the Lawrence Berkeley National Laboratory (LBNL). Datasets were indexed and scaled using HKL2000 (35) or XDS (36). Structures were determined by molecular replacement with the program PHASER (37) within the Phenix suite (38) using the Rosetta design models as the initial search model. The molecular replacement results were used to build the design structures and initiate crystallographic refinement and model rebuilding. Structure refinement was performed using the phenix.refine program (38, 39). Manual rebuilding using the COOT (Crystallographic Object-Oriented Toolkit) software program (40) and the addition of water molecules allowed construction of the final models. Summaries of statistics are provided in *SI Appendix*, Table S3.

### NMR Spectroscopy and Structure Determination.

All experiments were recorded at 37 °C on an 800-MHz Bruker Avance III spectrometer equipped with a triple resonance inverse (TCI) cryoprobe. NMR samples contained 20-mM sodium phosphate pH 6.2, 100-mM NaCl, 0.01% NaN_3_, and 1-U protease inhibitor mixture (Roche) in 95% H_2_O/5% D_2_O. For backbone and methyl assignments, labeled samples were prepared using well-established protocols (41, 42). Briefly, backbone assignments were performed on uniformly ^13^C/^15^N, perdeuterated protein samples using transverse relaxation optimized spectroscopy (TROSY)-based 3D experiments, HNCO, HNCA and HN(CA)CB. Side-chain methyl assignments were performed starting from the backbone assignments and using unambiguous nuclear Overhauser effect (NOE) connectivities observed in 3D C_M_-C_M_H_M_, 3D C_M_-NH_N_, and 3D H_N_H_α_-C_M_H_M_ band-Selective Optimized-Flip-Angle Short-Transient (SOFAST) nuclear Overhauser enhancement spectroscopy (NOESY) experiments, recorded on a U-[^15^N, ^12^C, ^2^H] MAI(LV)^proS^ (Met ^13^Cε, Ala ^13^Cβ, Ile ^13^Cδ1, Leu ^12^Cδ1/^13^Cδ2, Val ^12^Cγ1/^13^Cγ2) or AI(LV)^proS^ selective methyl-labeled sample. The calcium titrations for XAA_GVDQ were performed in 20-mM Tris⋅HCl pH 7.2, 100-mM NaCl, and 0-, 0.5-, 1-, 2-, 5-, 10-, 15-, 20-, 25-, 30-, and 50-mM CaCl_2_ buffer conditions.

Backbone amide ^1^D_NH_ RDCs were obtained using 2D ^1^H-^15^N transverse relaxation-optimized spectroscopy (ARTSY) (43). For the XAA_GVDQ sample, the alignment media were 10-mg/mL Pf1 phage (ASLA Biotech) for experiments performed without calcium and 15-mg/mL Pf1 phage for experiments performed in the presence of 5-mM CaCl_2_. The ^2^H quadrupolar splittings were 8.7 Hz and 9.3 Hz in the absence and presence of CaCl_2_, respectively. For the XAA sample, the alignment media were 10-mg/mL Pf1 phage and the ^2^H quadrupolar splitting was equal to 6.2 Hz. All NMR data were processed with NMRPipe (44) and analyzed using NMRFAM-SPARKY (45). The chemical shift deviations (CSDs) of ^13^C methyls (p.p.m.) were calculated using the equation Δδ^CH3^ = [1/2(Δδ_H_^2^ + Δδ_C_^2^/4)]^1/2^. The CSDs of ^15^N amides (p.p.m.) were calculated using the equation Δδ^NH^ = [1/2(Δδ_H_^2^ + Δδ_N_^2^/25)]^1/2^.

Structure modeling from our NMR data was performed using Rosetta’s fold-and-dock protocol (30, 46–48). Two sets of calculations were performed using different input data: 1) backbone chemical shifts and RDCs (to model the structure of XAA_GVDQ in the presence and absence of calcium [Fig. 4 *A*, *Bottom*]) and 2) backbone chemical shifts, long-range NOEs, and RDCs (to calculate the de novo NMR structures for XAA [Fig. 2*E*] and XAA_GVDQ [Fig. 4*D*]) (49). In the second set of calculations, we used a uniform upper distance bound of 10 Å for all 92 NOE restraints for XAA and for all 139 NOE restraints for XAA_GVDQ (*SI Appendix*, Table S5). The 10 lowest-energy structures from each calculation showing good agreement with the RDCs and chemical shifts and a minimum number of NOE violations were deposited in the PDB under accession codes 6O0I for XAA (BMRB ID 30574) and 6O0C for XAA_GVDQ mutant M4L (BMRB ID 30573).

### Data Availability.

The data for X-ray structures have been deposited in the Research Collaboratory for Structural Bioinformatics Protein Data Bank (RCSB PDB) (https://www.rcsb.org/) under the following PDB IDs: 6NX2 (AAA), 6NYI (XXA), 6NYE (XAX), 6NYK (XAX_GGDQ), 6NZ1 (XXA_GVDQ), 6NZ3 (XAA_GGHN), 6NXM (XAA_GVDQ), and 6NY8 (XAA_GVDQ with calcium). The data for NMR structures have been deposited in the RCSB PDB under PDB IDs 6O0I (XAA de novo) and 6O0C (XAA_GVDQ mutant M4L de novo without calcium), and in the Biological Magnetic Resonance Data Bank (BMRB) (http://www.bmrb.wisc.edu/) under BMRB IDs 30574 (XAA de novo) and 30573 (XAA_GVDQ mutant M4L de novo without calcium). All other data are included in the manuscript and *SI Appendix*.

## Supplementary Material

Supplementary File
