# Supplementary material for: Computational design of closely related proteins that adopt two well-defined but structurally divergent folds
Source: Proc Natl Acad Sci U S A. 2020 Mar 18;117(13):7208–15. doi: 10.1073/pnas.1914808117 (PMC7132107; doi:10.1073/pnas.1914808117)
Supplement: Supplementary File [file pnas.1914808117.sapp.pdf]

Supporting Information

Supporting Figures

Fig S1 | Round 1 design scheme
Fig S2 | Characterization of round 1 designs
Fig S3 | Comparison of round 2 to round 1 designs
Fig S4 | Characterization of round 2 designs
Fig S5 | Round 3 design scheme and comparison to round 2
Fig S6 | Characterization of round 3 designs
Fig S7 | Protein expression and nickel purification
Fig S8 | Detailed characterization of XAA
Fig S9 | Backbone and methyl NMR assignments for XAA (left) and XAA_GVDQ (right)
Fig S10 | Bromine coordination site in AAA
Fig S11 | Calcium binding by three aspartates from the PDB
Fig S12 | Nickel binding by three histidines from the PDB
Fig S13 | Zinc binding by three cysteines from the PDB
Fig S14 | B-factors and helical propensities of selected designs
Fig S15 | 1D methyl proton spectra for XAA_GVDQ with calcium
Fig S16 | Crystal structure of XAA_GVDQ without calcium (PDB ID 6nxm)
Fig S17 | NMR assignments for XAA_GVDQ and XAA_GVDQ mutant M4L

Supporting Tables

Table S1 | Amino acid sequence of protein designs tested
Table S2 | Protein crystallization conditions of structures reported.
Table S3 | X-ray crystallography data collection and refinement statistics
Table S4 | SAXS molecular weight measurements for select designs
Table S5 | NMR restraints and structural statistics for XAA structural ensembles
Table S6 | NMR restraints and structural statistics for k170 structural ensembles

Supporting Text

Tuning of the free energy difference between short and long states
Supporting References

Supporting Figures

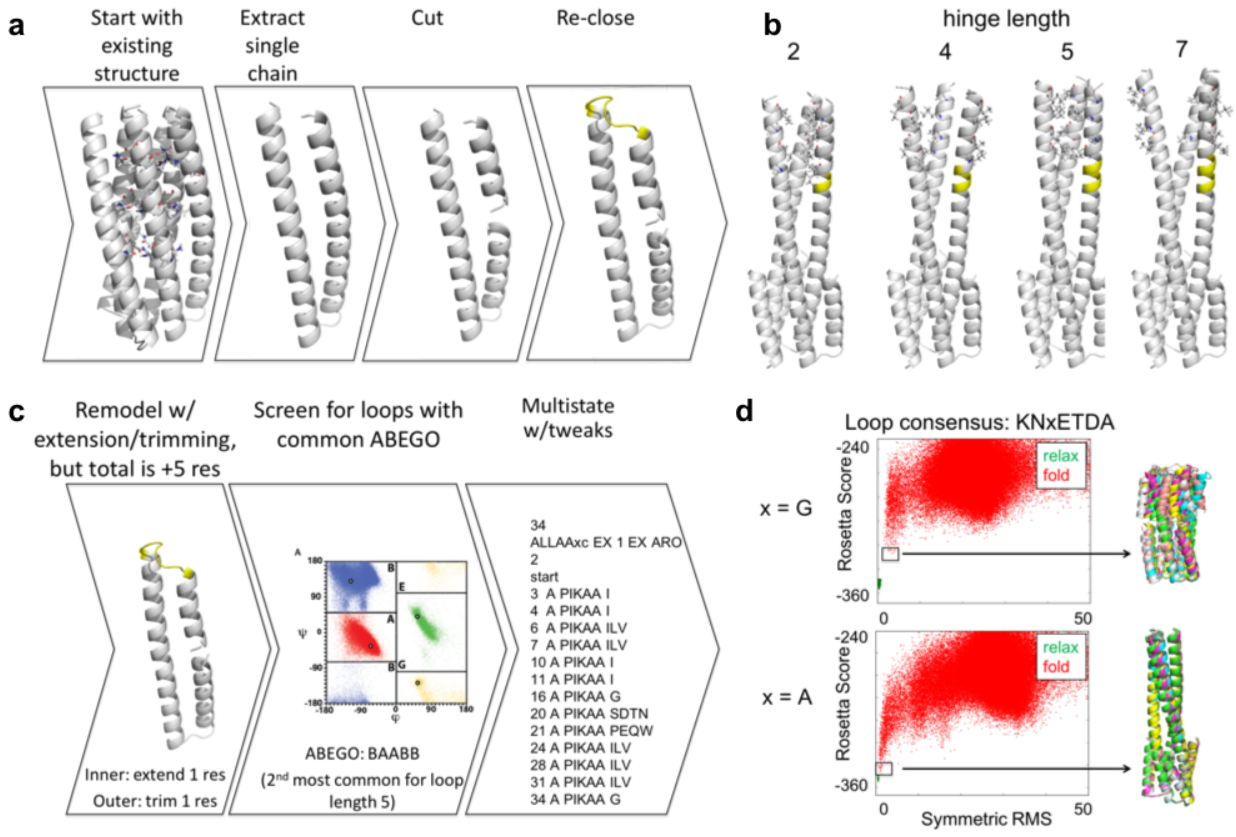

Fig S1 | Round 1 design scheme

a, Starting with previously characterized design 2L6HC3_13 variant (i.e., 2L6HC3_XAAA)(1), the protein is rearranged to form the desired architecture. Helix capping residues were incorporated at the N-terminus, and hydrogen bond networks within the flipping helix were redesigned using Rosetta to hydrophobic residues for stability. **b,** Different hinge lengths were tested for ability to pack in the long state. **c**, Loop closure using the determined hinge length. **d**, Testing for sequences that are predicted by Rosetta to fold in the short or long state.

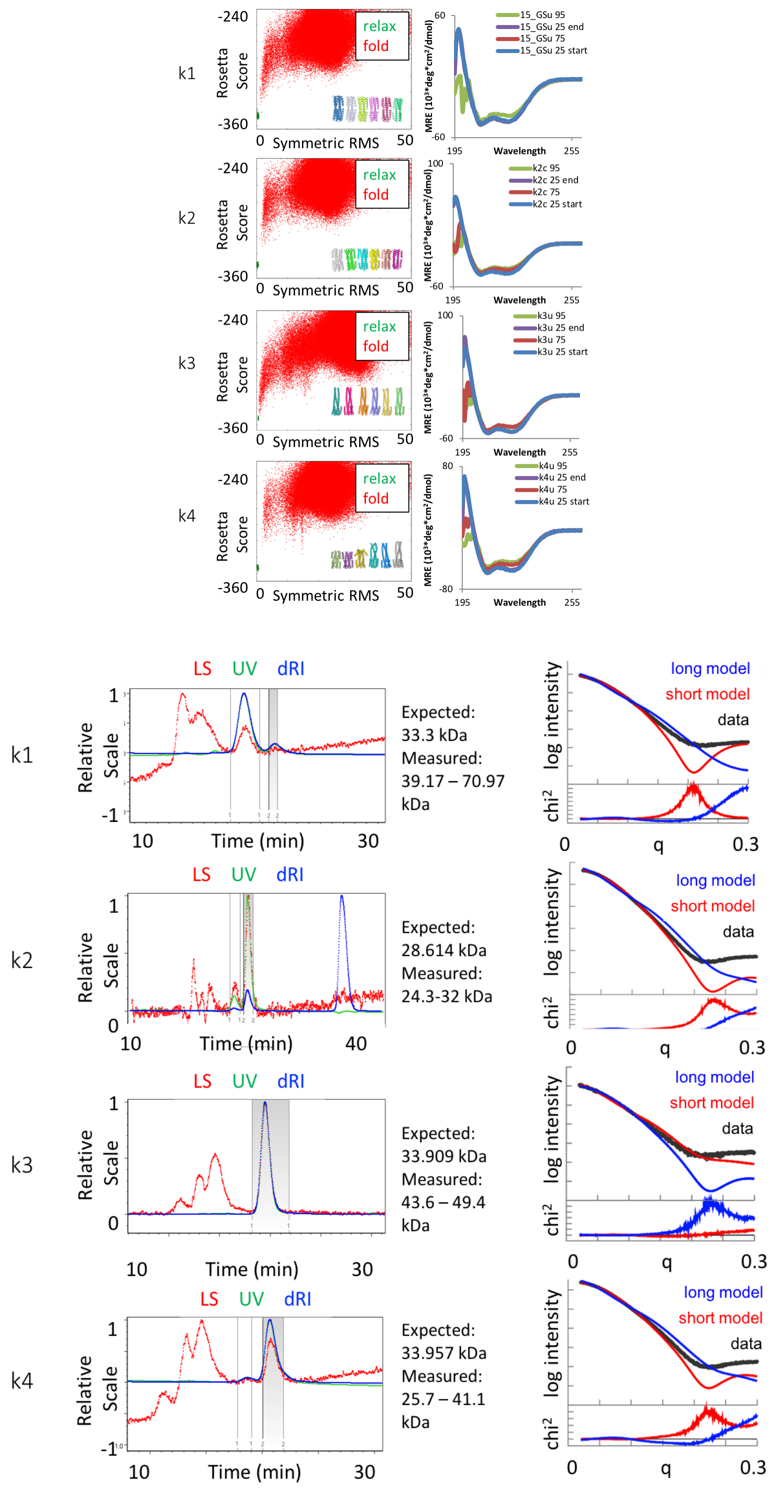

Fig S2 | Characterization of round 1 designs
(first column, top) Rosetta folding predictions, (second column, top) circular dichroism (CD) measurements, (first column, bottom) size exclusion chromatography with multi-angle light scattering (SEC-MALS) measurements, and (second column, bottom) small angle x-ray scattering (SAXS) measurements of designs k1-k4.

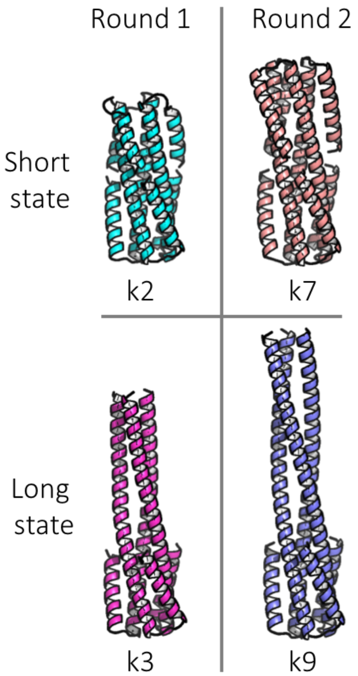

Fig S3 | Comparison of round 2 to round 1 designs
Round 2 designs have longer flipping helices, compared to round 1 designs, to facilitate discrimination by SAXS.

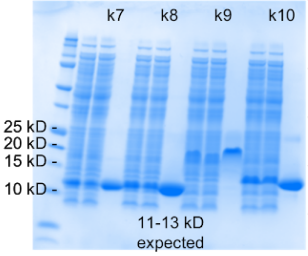

Name	Designed State	Expected monomer (kDa)	MS (kDa)	Expected trimer (kDa)	SEC-MALS (kDa)	SAXS (chi vs short)	SAXS (chi vs long)
k7	short	12.815	12.80	33.192	36 (+/- 26%)	2.02	5.03
K8	short	12.763	12.75	33.036	40 (+/- 22%)	3.12	5.06
k9	long	13.058	13.05	33.921	35 (+/- 13%)	4.03	1.98
k10	long	12.847	12.84	33.288	No signal	-	-

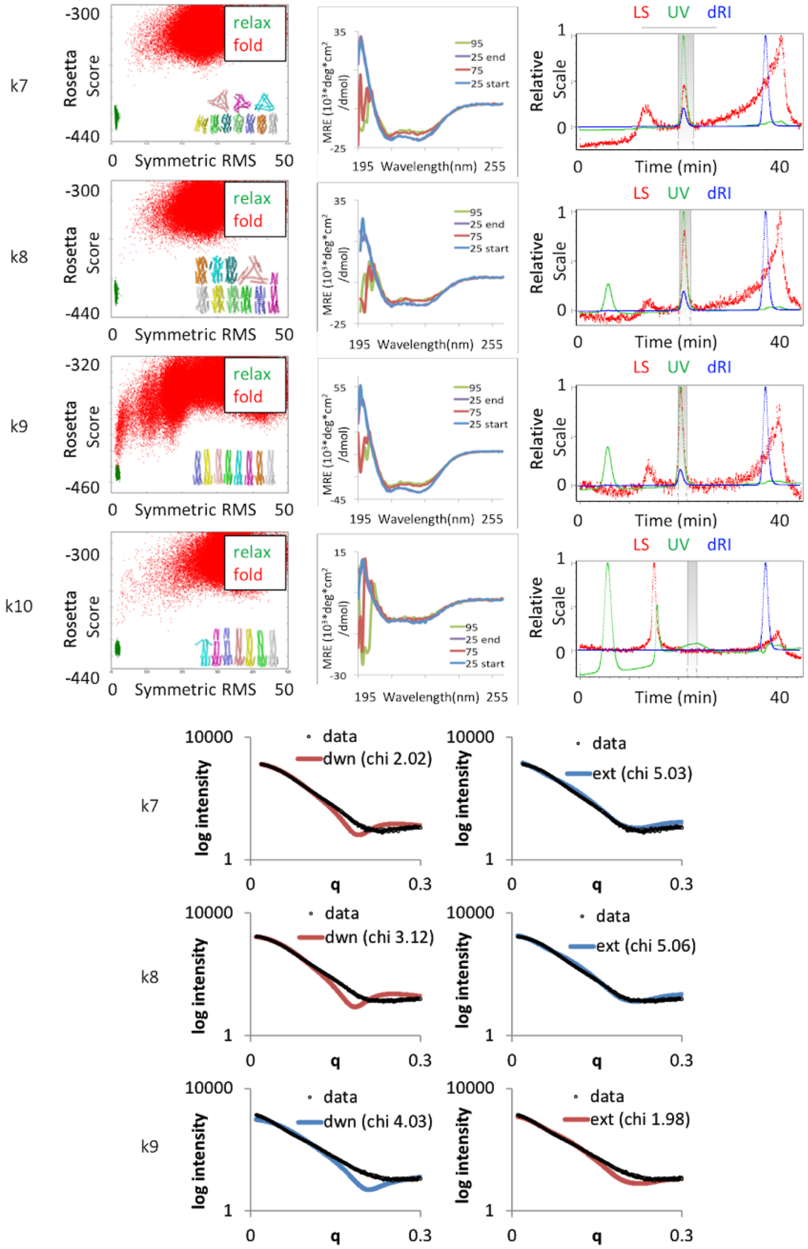

Fig S4 | Characterization of round 2 designs
(top) SDS-PAGE gel, table summary of characterization results, (first column) Rosetta folding predictions, (second column) CD measurements, (third column) SEC-MALS measurements, and (bottom columns) SAXS measurements of designs k7-10. Design k9 runs larger than expected on SDS-PAGE gels, but this can sometimes occur for highly helical proteins.

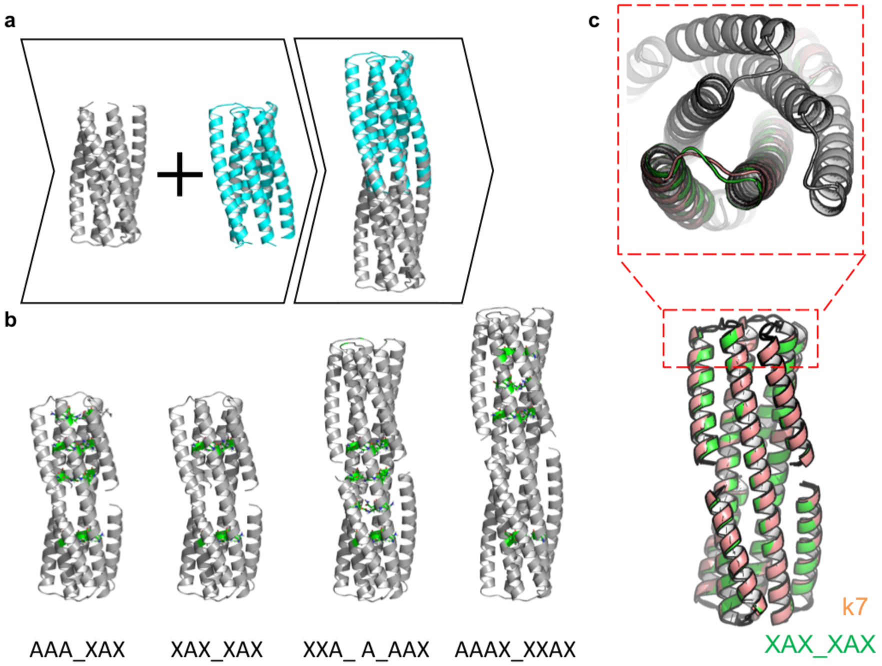

Fig S5 | Round 3 design scheme and comparison to round 2
a, Backbone construction by fusing 2L6HC3_13 variant (2L6HC3_XAAA) (grey) to 2L6HC3_23 (cyan)(1) via the inner three helices. Outer helices are trimmed to remove clashes and the exposed inner helices were redesigned using Rosetta to remove surface exposed hydrogen bond networks. **b**, Four variants with different fusion positions, placement of hydrogen bond networks (highlighted in green), and loop connectivity. **c,** Compared to the second generation designs (k7, orange), the flipping helix of round 3 design (XAX_XAX, green) is positioned slightly closer to the inner helices in the short state and the termini of the hinge are slightly closer together.

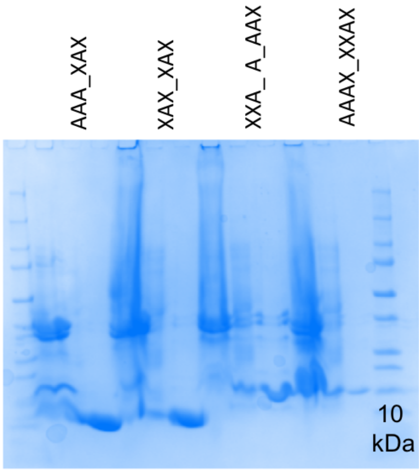

Name	Expected trimer (kDa)	SEC-MALS (kDa)
AAA_XAX	38.664	40.21 (+/-30.5)
XAX_XAX	38.301	41.4 (+/-1.763)
XXA_ A_AAX	49.578	40.3 (+/-1.9)
AAAX_XXAX	53.841	43.58 (+/-7.9)

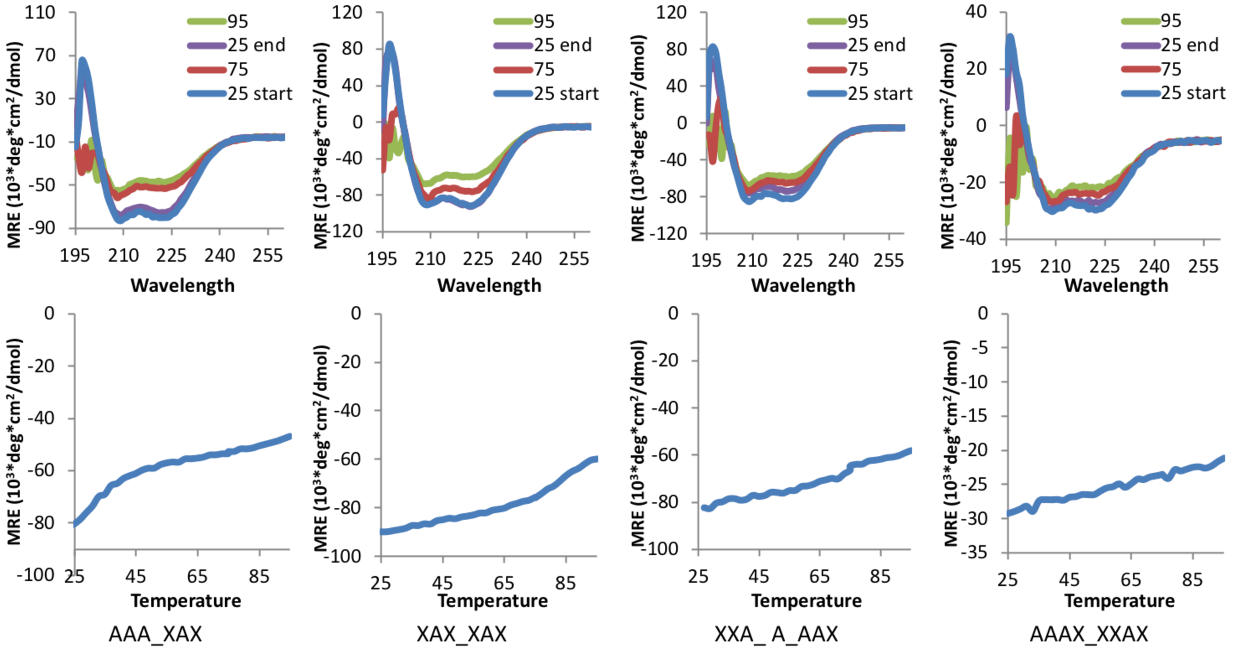

Fig S6 | Characterization of round 3 designs

(top) SDS-PAGE gel and table summary of characterization results, and (bottom) CD measurements for round 3 designs. Top row shows wavelength scans at 25ºC, 75ºC, 95ºC, and samples cooled back to 25ºC. Bottom row shows temperature melts of constructs from 25ºC to 95ºC. AAA_XAX is also referred to as AAA. Both XXA_A_AAX and AAAX_XXAX appear smaller than would be expected of trimers by SEC-MALS.

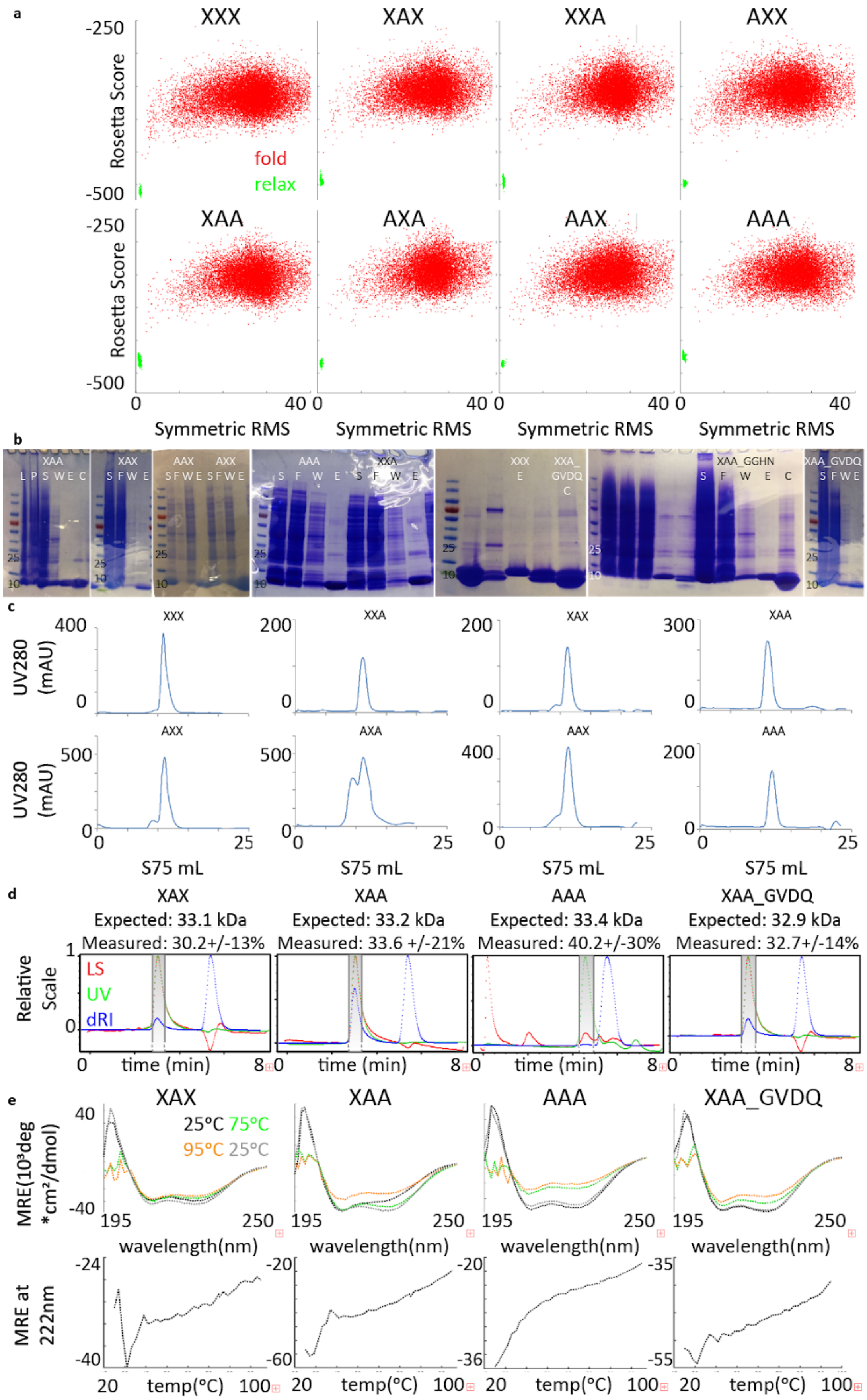
a
XXX
XAX
XXA
AXX
XAA
AXA
AAX
AAA
Rosetta Score
-250
-500
fold
relax
Symmetric RMS
0
40
b
XAA
XAX
AAX
AXX
AAA
XXA
XXX
XAA_GVDQ
XAA_GGHN
S F W E
25
10
c
XXX
XXA
XAX
XAA
AXX
AXA
AAX
AAA
UV280 (mAU)
S75 mL
0
25
d
XAX
Expected: 33.1 kDa
Measured: 30.2+/-13%
XAA
Expected: 33.2 kDa
Measured: 33.6 +/-21%
AAA
Expected: 33.4 kDa
Measured: 40.2+/-30%
XAA_GVDQ
Expected: 32.9 kDa
Measured: 32.7+/-14%
Relative Scale
LS
UV
dRI
time (min)
e
XAX
XAA
AAA
XAA_GVDQ
25°C 75°C
95°C 25°C
MRE(10³deg *cm²/dmol)
wavelength(nm)
195
250
MRE at 222nm
temp(°C)
20
100

Fig S7 | Protein expression and nickel purification
a, Rosetta folding predictions plotted with Rosetta energy on the y-axis and symmetric RMS to input model on the x-axis. Green points are relaxation runs on the model (of the short state only) and red points are individual folding trajectories (based on sequence alone). **b,** SDS-PAGE gel showing various steps during nickel purification to verify expression and purity of designs. L = cell lysate; P = pellet; S = supernatant; F = flow through; W = wash 1 (of 3); E = elution; C = thrombin cleaved. **c,** FPLC purification. Absorbance at 280 nm vs. column volume for protein samples run on a Superdex 75 10/300 GL column in 20 mM Tris, pH 8, 150 mM NaCl, 2% glycerol. Most samples were monodisperse or have a relatively small aggregation peak. AXA has a large soluble aggregate peak, but has a prominent peak at the expected position for a trimer. **d,** SEC-MALS measurements. Expected mass is calculated for trimers after thrombin cleavage to remove histidine purification tag. **e,** CD measurements. (top) wavelength scans at 25ºC, 75ºC, 95ºC, and samples cooled back to 25ºC and (bottom) temperature melts of constructs from 25ºC to 95ºC.

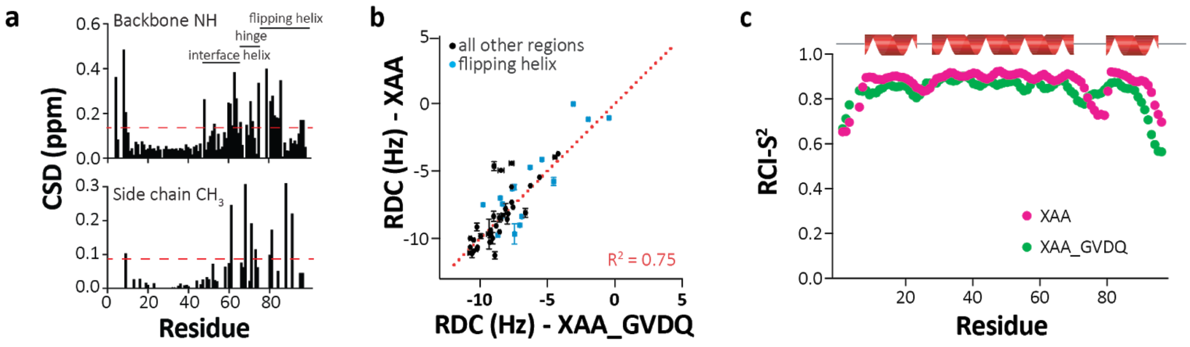

Fig S8 | Detailed characterization of XAA
a, Chemical shift deviations (CSDs) for amides and methyls shared between XAA and XAA_GVDQ show differences in the flipping helix interface to the inner helices. **b,** RDC experiments for XAA vs XAA_GVDQ. **c,** Estimated backbone order parameter (Random Coil Index S^2) derived from the chemical shifts(2) is shown for XAA and XAA_GVDQ residues. Lower RCI-S^2 indicates flexibility, higher RCI-S^2 indicates rigidity. Secondary Structure Index (SSI), predicting three helical segments here, are derived from TALOS-N analysis of backbone ($^{13}C^{a}$, $^{13}C'$, ^{15}N, $^{1}H^{a}$ and $^{1}H^{N}$) and $^{13}C^{b}$ chemical shifts.

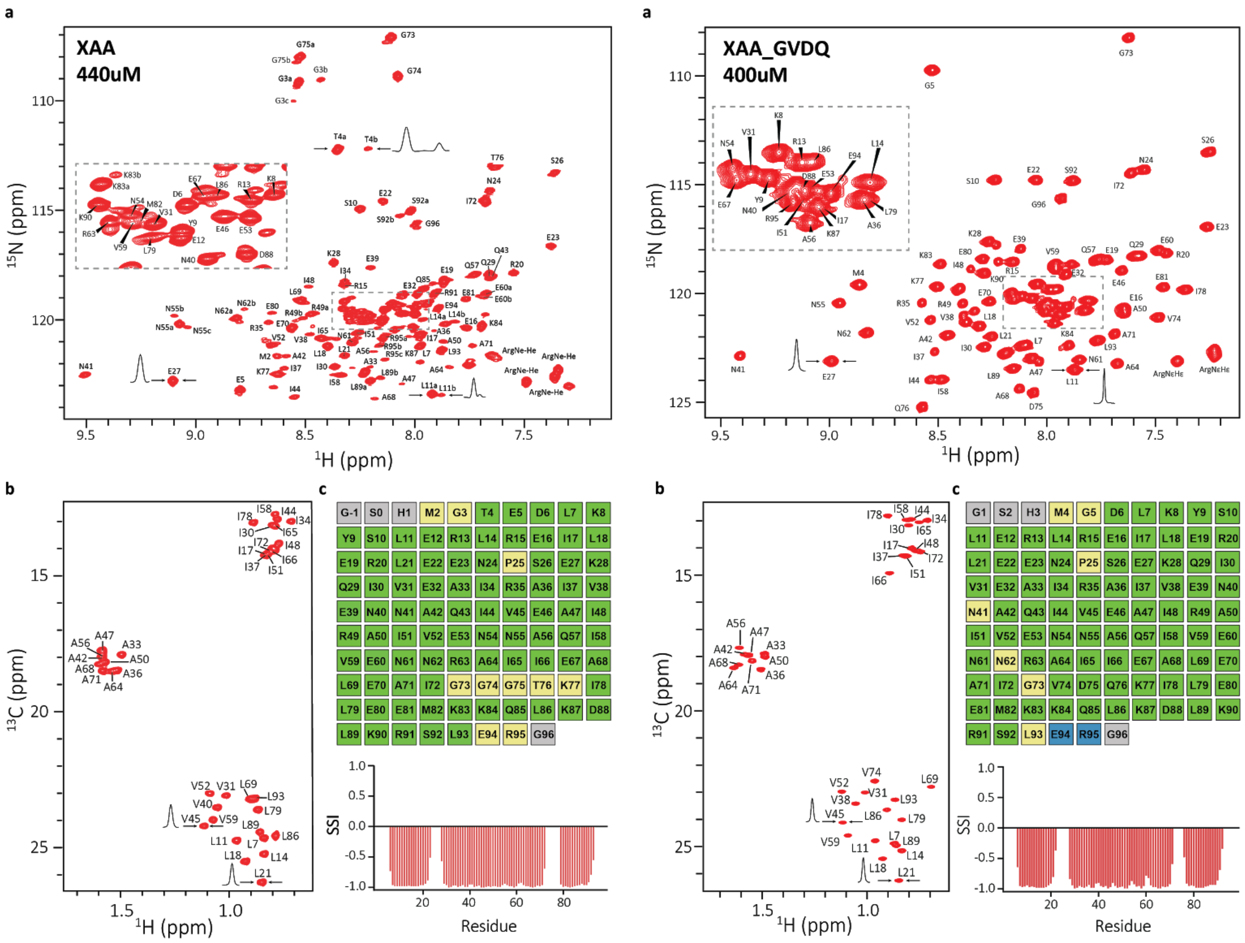

Fig S9 | Backbone and methyl NMR assignments for XAA (left) and XAA_GVDQ (right)
a, Fully assigned 2D ^{1}H-^{15}N TROSY-HSQC spectrum. 1D slices are shown with arrows in both spectra to indicate the line broadening on XAA and the presence of a minor conformation in various residues distributed to the entire structure of XAA which turns to be a heterogeneity. **b,** Fully assigned 2D ^{1}H-^{13}C HMQC for AI(LV)^{proS} samples. **c,** (top) TALOS-N validation data for backbone assignments. Assignment validation, green: highly confident assignments. yellow: acceptable within limits. blue: dynamic residues. grey: no classification. (bottom) Secondary Structure Index (SSI) derived from TALOS-N analysis of backbone (^{13}C$_a$, ^{13}C', ^{15}N, ^{1}H$_a$ and ^{1}H$_N$) and ^{13}C$_b$ chemical shifts. Positive SSI values are consistent with β-strand and negative values are consistent with α helical structure.

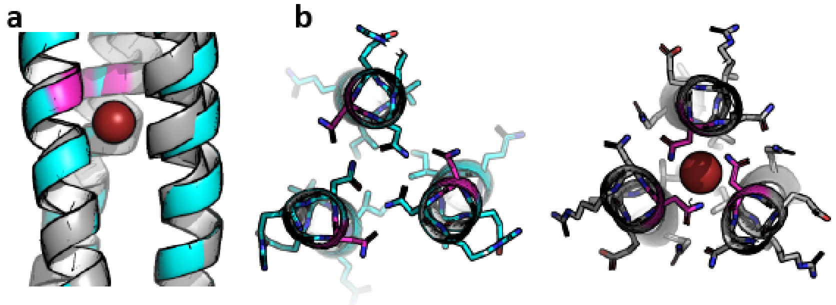

Fig S10 | Bromine coordination site in AAA
Model (cyan) and crystal (grey) crystal structure of AAA is shown near N61 (magenta), where there is deviation between the model (left) and the crystal due to the presence of bromine (right).

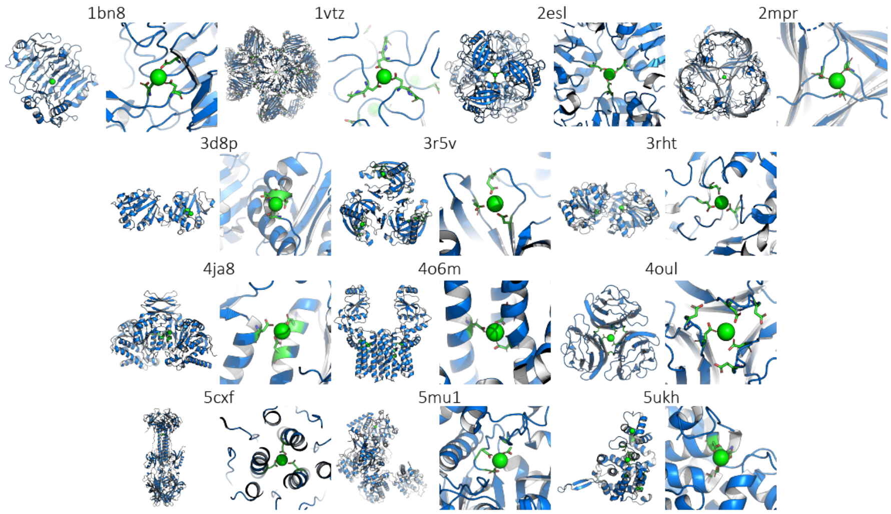

Fig S11 | Calcium binding by three aspartates from the PDB
The MetalPDB database (http://metalweb.cerm.unifi.it/search/metal/) was used to curate 13 structures with calcium coordinated by three aspartic acids (of 4520 labeled as calcium binding). For each structure, the PDB ID is given at the top, the full structure shown on the left, and a close up of the binding site shown on the right. Calcium and coordinating aspartates are shown in green.

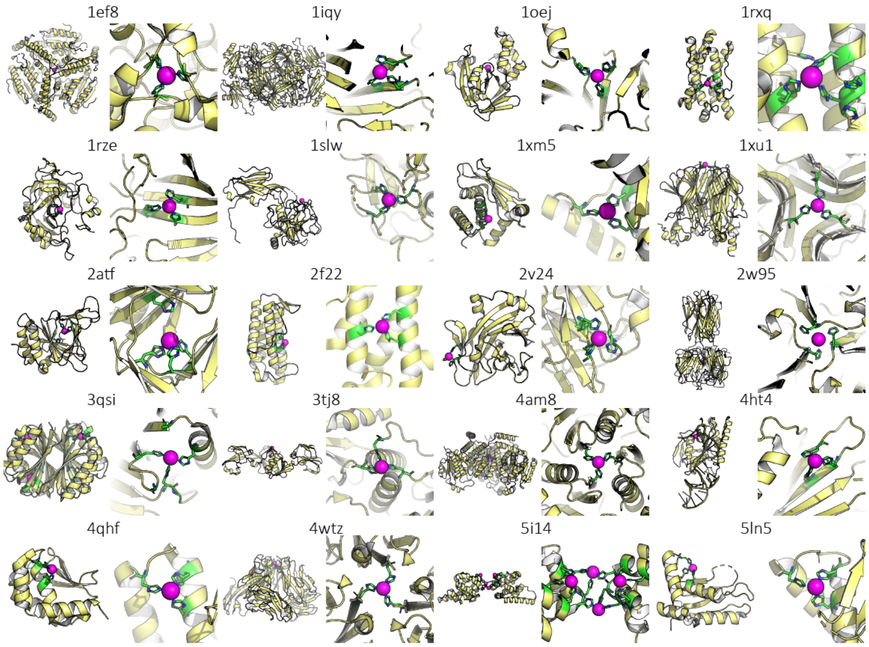

Fig S12 | Nickel binding by three histidines from the PDB
The MetalPDB database (http://metalweb.cerm.unifi.it/search/metal/) was used to curate 20 structures with zinc coordinated by three histidines (of 861 labeled as nickel binding). For each structure, the PDB ID is given at the top, the full structure shown on the left, and a close up of the binding site shown on the right. Nickel is shown in magenta and coordinating histidines are shown in green.

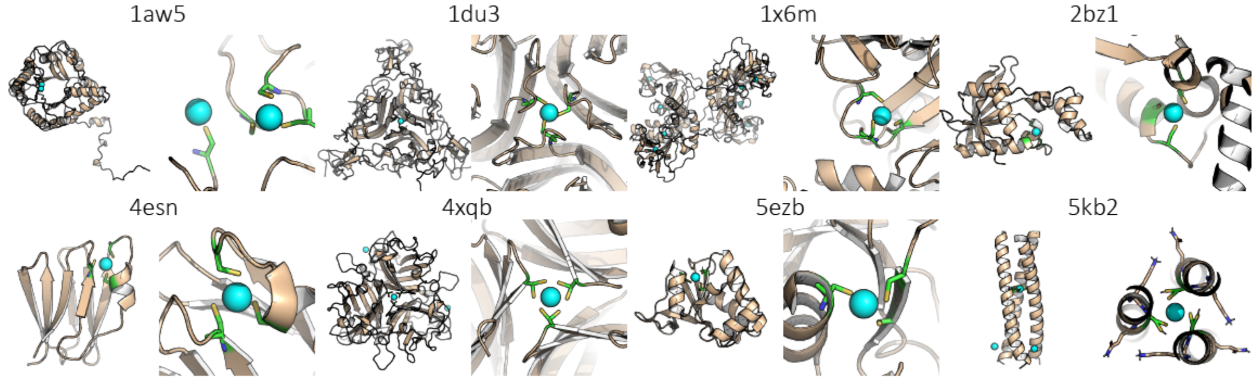

Fig S13 | Zinc binding by three cysteines from the PDB
The MetalPDB database (http://metalweb.cerm.unifi.it/search/metal/) was used to curate 8 structures with zinc coordinated by three cysteines (of 5467 labeled as zinc binding). For each structure, the PDB ID is given at the top, the full structure shown on the left, and a close up of the binding site shown on the right. Zinc is shown in cyan and coordinating cysteines are shown in green.

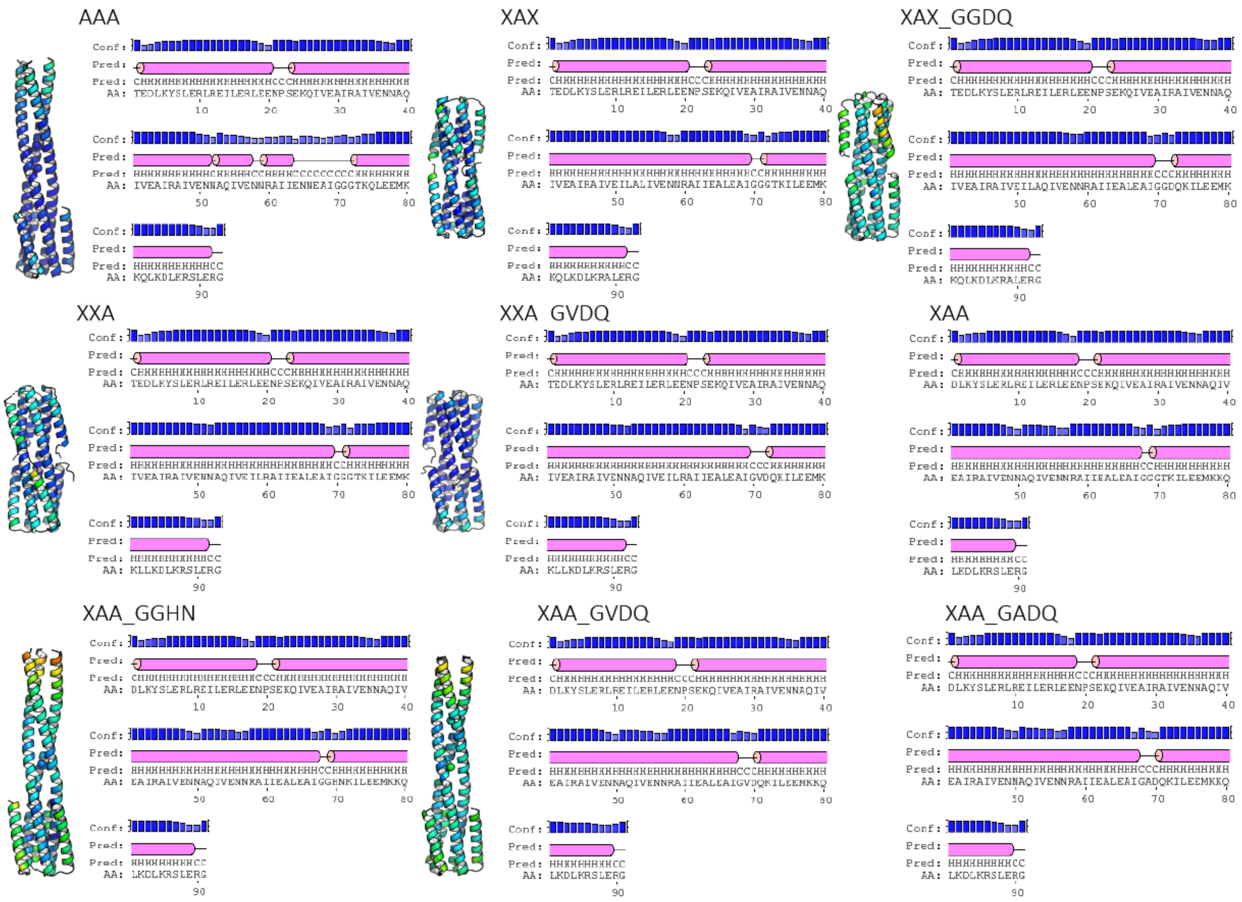

Fig S14 | B-factors and helical propensities of selected designs
B-factors are show on ribbon diagrams by color (blue is low, red is high; not normalized across structures). PSIPRED predictions are shown diagrammatically and by letter designation (3). Note that the numbering used in secondary structure prediction starts at 1 with the sequence shown and is offset from numbering of residues used in the main text (assigned to keep NMR, crystal, and model numbering in sync).

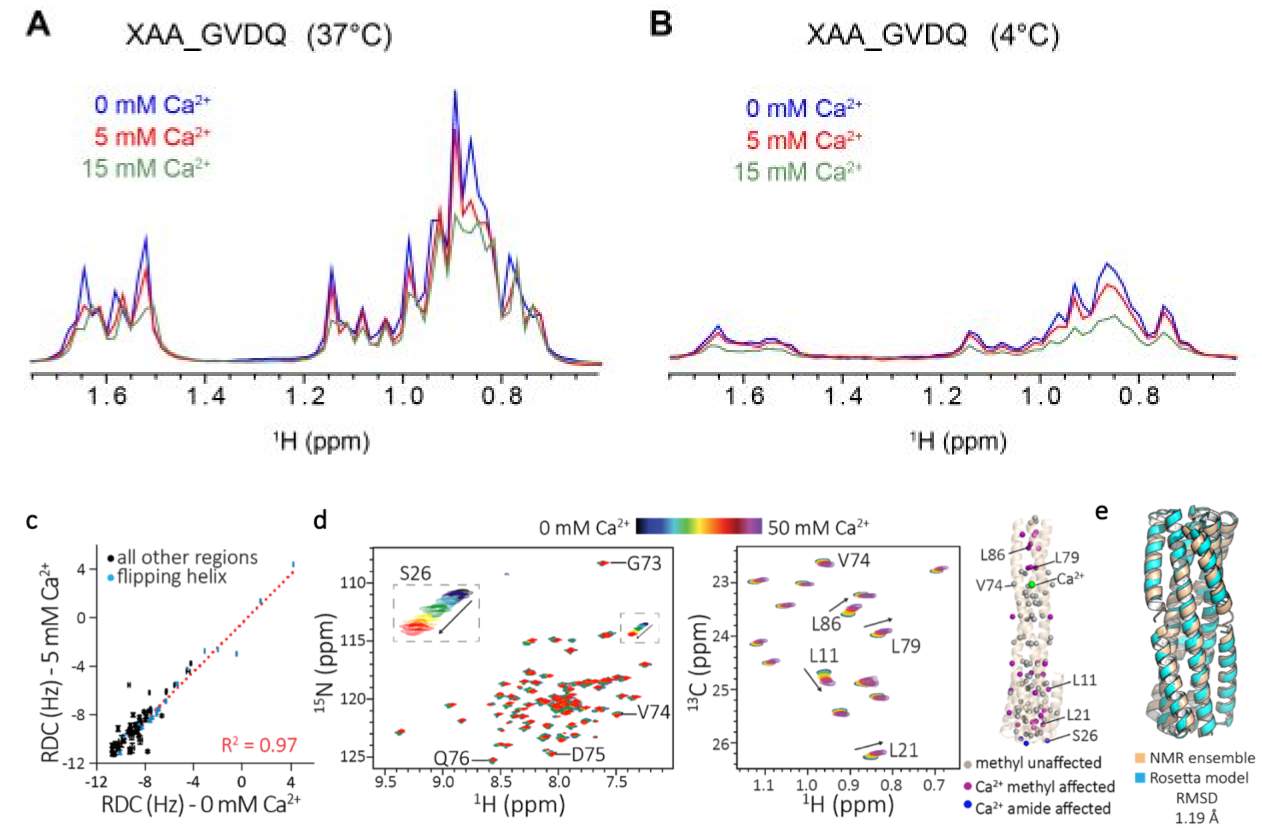

Fig S15 | 1D methyl proton spectra for XAA_GVDQ with calcium

Comparison of 1D projections along the ^1H dimension extracted from 2D ^1H-^{13}C methyl SOFAST HMQC experiments recorded at **a,** 37 °C or **b,** 4°C at a ^1H field strength of 800 MHz. For experiments at 37 °C, acquisition parameters were 4 scans/FID with a recycle delay of 0.2 sec and 128 / 1024 complex points in the ^{13}C / ^1H dimensions. For experiments at 4 °C, acquisition parameters were 8 scans/FID with a recycle delay of 0.2 sec and 128 / 1024 complex points in the ^{13}C / ^1H dimensions. Each experiment was recorded in the presence of varying concentrations of calcium using 105 µM MAI(LV)^{proS} labeled XAA_GVDQ in NMR buffer (100 mM NaCl, 20 mM Tris pH 7.2, 2% (v/v) d_8-glycerol). **c,** RDC experiments with 0 mM or 5 mM calcium. Amide groups corresponding to the C-terminal helix are colored blue. **d,** Calcium titration of XAA_GVDQ construct. (left) Overlay of 2D ^1H-^{15}N TROSY-HSQC spectra and (center) 2D ^1H-^{13}C HMQC spectra of AI(LV)^{proS} sample in different calcium concentrations (0-50 mM). Non-specific calcium binding is observed. G73, V74, D75 and Q76 are the residues expected in calcium binding and S26, L11, L21, L79 and L89 are the residues observed. (right) Distribution of calcium unaffected methyl (grey), methyl affected (magenta), and calcium amide affected (blue) residues overlaid on the long conformation expected from crystallographic data. **e**, De novo NMR structure for XAA_GVDQ (beige, PDB ID 6o0c) compared to design model (cyan) confirms the protein is in the compact conformation (RMSD = 1.19 Å).

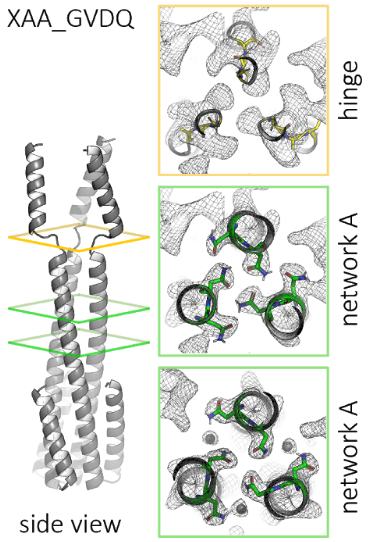

Fig S16 | Crystal structure of XAA_GVDQ without calcium (PDB ID 6nxm)
The protein crystallizes as a domain swapped hexamer (only trimer shown) with contacts in the flipping helix replicating the contacts of the short model state, but with another molecule of XAA_GVDQ rather than with self. Details showing the GVDQ hinge region forms a very flexible linker that allows the domain swap. Designed hydrogen bond networks remain intact.

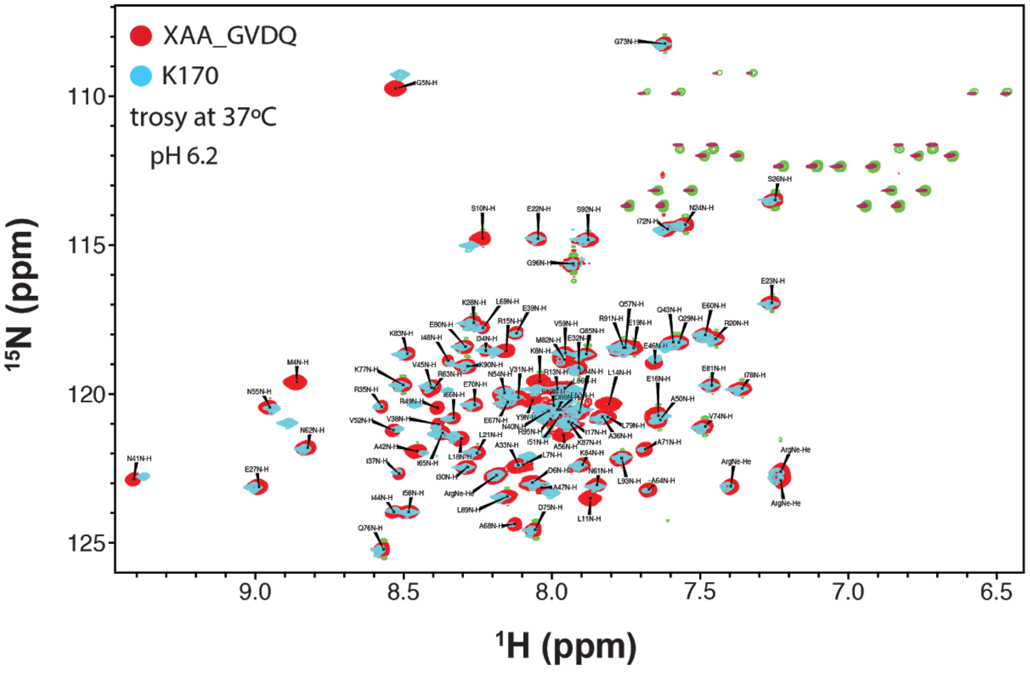

Fig S17 | NMR assignments for XAA_GVDQ and XAA_GVDQ mutant M4L
TROSY showing that XAA_GVDQ and k170 (a single point mutant of XAA_GVDQ with M4L designed to facilitate NMR experiments) show the two structures are identical except at the M4 and G5.

Supporting Tables

Table S1 | Amino acid sequence of protein designs tested in fasta format
Rosetta designs begin two residues after the thrombin cleavage and NdeI cut site of the pET28b backbone (LVPRGSHM). The glycine after the NdeI cut site is introduced manually to provide flexible linkage to the preceding histidine tag. Thrombin cleavage removes the N-terminal tag up the apostrophe in the sequence LVPR'GS. k170 is a point mutant of XAA_GVDQ designed to remove the methionine from the plasmid backbone to facilitate NMR characterization and behaves identically to XAA_GVDQ in NMR experiments (Fig. S15). Alternative names are given in parenthesis)

>k1 (k1_15_GSGSGdwn)
MGSSHHHHHHSSGLVPRGSHMGSELKYSLERLREILERLEENPSEDVIVEAIRAIVENNK
QIVEAIREIIEVIEKIIRALGSGSGDAREIEKAVREVKKG

>k2 (k2_15_KNGETdwn)
MGSSHHHHHHSSGLVPRGSHMGSELKYSLERLREILERLEENPSEDVIVEAIRAIVENNK
QIVEAIREIIEVIEKIIRALKNGETDAREIEKAVREVKKG

>k3 (k3_15_KNAEText)
MGSSHHHHHHSSGLVPRGSHMGSELKYSLERLREILERLEENPSEDVIVEAIRAIVENNK
QIVEAIREIIEVIEKIIRALKNAETDAREIEKAVREVKKG

>k4 (k4_15_KNSETdwn)
MGSSHHHHHHSSGLVPRGSHMGSELKYSLERLREILERLEENPSEDVIVEAIRAIVENNK
QIVEAIREIIEVIEKIIRALKNSETDAREIEKAVREVKKG

>k7 (cla32_dwn)
MGSSHHHHHHSSGLVPRGSHMGSEDLKYSLERLREILECLEENPSEKQIVEAIRAIVENN
KQIVEAIKKILEILKLLAKNNKLVADILRELGVSPELLDELEKSLRELERMLQ

>k8 (cla32_GS_dwn)
MGSSHHHHHHSSGLVPRGSHMGSEDLKYSLERLREILECLEENPSEKQIVEAIRAIVENN
KQIVEAIKKILEILKLLAKNNKLVADILRELGSGSELLDELEKSLRELERMLQ

>k9 (cla32_ext)
MGSSHHHHHHSSGLVPRGSHMGSEDLKYSLERLREILECLEENPSEKQIVEAIRAIVENN
KQIVEAIKRILDELQKIKEEIRKIKDEIAKIKEEIEKIKREIAKIKEEIKKAL

>k10 (cla32_GS_ext)
MGSSHHHHHHSSGLVPRGSHMGSEDLKYSLERLREILECLEENPSEKQIVEAIRAIVENN
KQIVEAIKRILDELQKIKEEIRKIKDEIAKIGSGSEKIKREIAKIKEEIKKAL

>XAX_XAX (k25)
MGSSHHHHHHSSGLVPRGSHMGTEDLKYSLERLREILERLEENPSEKQIVEAIRAIVENN
AQIVEAIRAIVETLALIVENNRAIIEWCEAVGGGTKILEEMKKQLKDLKRALERG

>XXA_A_AAX (k27)
MGSSHHHHHHSSGLVPRGSHMGSQLEEMKKQLYDLKRSLERLREILERLEENPSEKQIV
EAIRAIVENNKQIVENNRSIIENNEIIVKNNEIIVKVLSVIAEVLKIIAKILENPSEYMLKELK
KALKELEKMLKELRKSLKEL

>AAAX_XXAX (k28)
MGSSHHHHHHSSGLVPRGSHMGNEEARKTLEEMKKALKDLKRSLERLREILERLEENPS
EKQIVESIRSIVENNAQIVEVLARIIEALAIIVELIRKIVENNAQIVENNASIIENNATIIRALE
NPSEYTLDEARKQLEEMKKQLKDLKRSLERLRG

>AAA (k24)
MGSSHHHHHHSSGLVPRGSHMGTEDLKYSLERLREILERLEENPSEKQIVEAIRAIVENN
AQIVEAIRAIVENNAQIVENNRAIIENNEAIGGGTKQLEEMKKQLKDLKRSLERG

>AAX (k125)
MGSSHHHHHHSSGLVPRGSHMGTEDLKYSLERLREILERLEENPSEKQIVEAIRAIVENN
AQIVEAIRAIVEILALIVENNRAIIENNEAIGGGTKQLEEMKKQLKDLKRALERG

>AXA (k155)
MGSSHHHHHHSSGLVPRGSHMGTEDLKYSLERLREILERLEENPSEKQIVEAIRAIVENN
AQIVEAIRAIVENNAQIVEILRAIIENNEAIGGGTKQLEEMKKLLKDLKRSLERG

>AXX (k126)
MGSSHHHHHHSSGLVPRGSHMGTEDLKYSLERLREILERLEENPSEKQIVEAIRAIVENN
AQIVEAIRAIVEILALIVEILRAIIENNEAIGGGTKQLEEMKKLLKDLKRALERG

>XAA (k53)
MGSSHHHHHHSSGLVPRGSHMGTEDLKYSLERLREILERLEENPSEKQIVEAIRAIVENN
AQIVEAIRAIVENNAQIVENNRAIIEALEAIGGGTKILEEMKKQLKDLKRSLERG

>XXA (k127)
MGSSHHHHHHSSGLVPRGSHMGTEDLKYSLERLREILERLEENPSEKQIVEAIRAIVENN
AQIVEAIRAIVENNAQIVEILRAIIEALEAIGGGTKILEEMKKLLKDLKRSLERG

>XAX (k55)
MGSSHHHHHHSSGLVPRGSHMGTEDLKYSLERLREILERLEENPSEKQIVEAIRAIVENN
AQIVEAIRAIVEILALIVENNRAIIEALEAIGGGTKILEEMKKQLKDLKRALERG

>XXX (k156)
MGSSHHHHHHSSGLVPRGSHMGTEDLKYSLERLREILERLEENPSEKQIVEAIRAIVENN
AQIVEAIRAIVEILALIVEILRAIIEALEAIGGGTKILEEMKKLLKDLKRALERG

>XAX_GGDQ (k151)
MGSSHHHHHHSSGLVPRGSHMGTEDLKYSLERLREILERLEENPSEKQIVEAIRAIVENN
AQIVEAIRAIVEILAQIVENNRAIIEALEAIGGDQKILEEMKKQLKDLKRALERG

>XXA_GVDQ (k158)
MGSSHHHHHHSSGLVPRGSHMGTEDLKYSLERLREILERLEENPSEKQIVEAIRAIVENN
AQIVEAIRAIVENNAQIVEILRAIIEALEAIGVDQKILEEMKKLLKDLKRSLERG

>XAA_GADQ (k178)
MGSSHHHHHHSSGLVPRGSHLGDLKYSLERLREILERLEENPSEKQIVEAIRAIVENNAQI
VEAIRAIVENNAQIVENNRAIIEALEAIGADQKILEEMKKQLKDLKRSLERG

>XAA_GGHN (k167)
MGSSHHHHHHSSGLVPRGSHMGDLKYSLERLREILERLEENPSEKQIVEAIRAIVENNAQ
IVEAIRAIVENNAQIVENNRAIIEALEAIGGHNKILEEMKKQLKDLKRSLERG

>XAA_GVDQ (k54)
MGSSHHHHHHSSGLVPRGSHMGDLKYSLERLREILERLEENPSEKQIVEAIRAIVENNAQ
IVEAIRAIVENNAQIVENNRAIIEALEAIGVDQKILEEMKKQLKDLKRSLERG

>k170
MGSSHHHHHHSSGLVPRGSHLGDLKYSLERLREILERLEENPSEKQIVEAIRAIVENNAQI
VEAIRAIVENNAQIVENNRAIIEALEAIGVDQKILEEMKKQLKDLKRSLERG

Table S2 | Protein crystallization conditions of structures reported.

Design Name	Screen	Crystallization condition	Temp
AAA	Morpheus B10	10% w/v PEG 8000, 20% v/v ethylene glycol, 0.03 M of each halide (sodium fluoride, sodium bromide, sodium iodide), 0.1 M bicine/Trizma base pH 8.5	4°C
XXA	JCSG Core III E7	0.16 M magnesium acetate, 0.08 M sodium cacodylate pH 6.5, 20% (v/v) glycerol	18°C
XAX	JCSG Core I C1	0.2 M ammonium acetate, 20% (w/v) PEG 3350	4°C
XAX_GGDQ	JCSG Core II E3	1.0 M lithium chloride, 0.1 M MES pH 6.0, 20% (w/v) PEG 6000	18°C
XXA_GVDQ	Morpheus A3	10% w/v PEG 4000, 20% v/v glycerol, 0.03 M of each divalent cation (magnesium chloride, calcium chloride), 0.1 M MES/imidazole pH 6.5	18°C
XAA_GGHN	JCSG Core IV A3	0.1 M CAPS pH 10.5, 40% (v/v) MPD	18°C
XAA_GVDQ	JCSG Core III H1	2.0 M sodium formate, 0.1 M sodium acetate pH 4.6	18°C
XAA_GVDQ_ calcium	JCSG Core IV G12	0.05 M calcium acetate, 0.1 M sodium acetate pH 4.5, 40% (w/v) 1,2-propanediol	18°C

Table S3 | X-ray crystallography data collection and refinement statistics

Except for AAA (sent frozen to ALS 8.2.1), all other crystals were looped and frozen at the beamline (ALS 8.3.1). Beamline recommended strategies for collection was used. All structures suffered from poor diffraction data, some degree of radiation damage, potentially incorrect space group determination, and/or tNCS, resulting in higher R-free than typically expected of 1.9-2.8 Å resolution datasets. In regards to space group, automatic processing by XDS almost always determined the space group to be H32, which was incorrect. Reprocessing in other space groups (C2, P2, and P1) was tested, but not exhaustively.

Design Name<br>Biological assembly<br>PDB ID	AAA<br>trimer<br>6NX2	XXA<br>trimer<br>6NYI	XAX<br>trimer<br>6NYE
Resolution range	35.93 - 2.303 (2.385 - 2.303)	63.18 - 2.3 (2.382 - 2.3)	48.47 - 1.9 (1.968 - 1.9)
Space group	C 1 2 1	C 1 2 1	P 21 21 21
Unit cell	89.592 51.65 85.527 90 110.485 90	129.403 51.213 48.838 90 102.447 90	35.179 75.322 96.944 90 90 90
Total reflections	58747 (4463)	93511 (8762)	251950 (15068)
Unique reflections	16161 (1486)	14041 (1384)	21012 (2046)
Multiplicity	3.6 (3.0)	6.7 (6.3)	12.0 (7.4)
Completeness (%)	90.14 (69.63)	92.10 (82.28)	96.77 (87.78)
Mean I/sigma(I)	9.42 (1.32)	10.01 (1.29)	14.25 (0.85)
Wilson B-factor	55.42	32.95	26.59
R-merge	0.0686 (0.7771)	0.137 (1.532)	0.1199 (1.974)
R-meas	0.08064 (0.9321)	0.1487 (1.67)	0.1252 (2.125)
R-pim	0.04202 (0.5077)	0.05723 (0.6565)	0.03571 (0.768)
CC1/2	0.996 (0.709)	0.999 (0.737)	0.999 (0.378)
CC*	0.999 (0.911)	1 (0.921)	1 (0.74)
Reflections used in refinement	16161 (1137)	14041 (1142)	21012 (1818)
Reflections used for R-free	1487 (111)	1312 (114)	1703 (150)
R-work	0.2594 (0.3944)	0.2588 (0.4285)	0.2019 (0.3513)
R-free	0.2791 (0.4538)	0.2810 (0.4488)	0.2335 (0.3626)
CC(work)	0.942 (0.746)	0.967 (0.817)	0.971 (0.668)
CC(free)	0.927 (0.663)	0.958 (0.602)	0.932 (0.637)
Number of non-hydrogen atoms	1957	2107	2317
macromolecules	1944	2077	2141
ligands	2		
solvent	11	30	176
Protein residues	267	286	283
RMS(bonds)	0.005	0.004	0.005
RMS(angles)	0.51	0.79	0.81
Ramachandran favored (%)	99.62	98.19	100
Ramachandran allowed (%)	0.38	1.81	0
Ramachandran outliers (%)	0	0	0
Rotamer outliers (%)	6.86	1.04	0.97
Clashscore	1.61	4.33	2.08
Average B-factor	88.09	51.87	31.79
macromolecules	88.14	51.91	31.42
ligands	68.44		
solvent	82.61	49.08	36.26
Number of TLS groups	3		

Design Name<br>Biological assembly<br>PDB ID	XAX_GGDQ<br>trimer<br>6NYK	XXA_GVDQ<br>trimer<br>6NZ1	XAA_GGHN<br>trimer<br>6NZ3
Resolution range	45.34 - 2.8 (2.9 - 2.8)	88.36 - 1.9 (1.968 - 1.9)	43.67 - 2.3 (2.382 - 2.3)
Space group	C 1 2 1	P 1 21 1	C 1 2 1
Unit cell	95.416 55.002 57.906 90 123.381 90	50.647 76.747 91.946 90 106.064 90	88.741 51.271 85.873 90 110.076 90
Total reflections	41579 (4425)	355141 (36439)	107072 (10366)
Unique reflections	6209 (631)	53261 (5304)	16278 (1626)
Multiplicity	6.7 (7.0)	6.7 (6.9)	6.6 (6.4)
Completeness (%)	95.12 (90.73)	99.70 (99.77)	98.15 (96.20)
Mean I/sigma(I)	10.20 (1.40)	8.98 (1.46)	31.70 (8.63)
Wilson B-factor	82.44		47.9
R-merge	0.08044 (1.306)	0.1542 (1.073)	0.03 (0.1729)
R-meas	0.08715 (1.409)	0.168 (1.161)	0.03272 (0.1885)
R-pim	0.03319 (0.5248)	0.06572 (0.4397)	0.01288 (0.07428)
CC1/2	0.999 (0.609)	0.995 (0.703)	1 (0.996)
CC*	1 (0.87)	0.999 (0.908)	1 (0.999)
Reflections used in refinement	6209 (587)	53261 (5304)	16278 (1571)
Reflections used for R-free	607 (46)	1563 (162)	926 (93)
R-work	0.2617 (0.2997)	0.1847 (0.3347)	0.2620 (0.2687)
R-free	0.2971 (0.2876)	0.2159 (0.3631)	0.3017 (0.3071)
CC(work)	0.974 (0.760)	0.883 (0.697)	0.961 (0.911)
CC(free)	0.944 (0.877)	0.839 (0.413)	0.917 (0.793)
Number of non-hydrogen atoms	1729	4574	2041
macromolecules	1729	4317	2030
ligands			1
solvent		257	10
Protein residues	271	569	275
RMS(bonds)	0.002	0.009	0.004
RMS(angles)	0.41	0.9	0.54
Ramachandran favored (%)	98.85	97.49	99.63
Ramachandran allowed (%)	1.15	2.51	0.37
Ramachandran outliers (%)	0	0	0
Rotamer outliers (%)	0.86	0.71	0
Clashscore	6.65	6.1	6.27
Average B-factor	79.97	29.48	63.99
macromolecules	79.97	29.27	64.01
ligands			56.69
solvent		33.13	60.62
Number of TLS groups		17	

Design Name<br>Biological assembly<br>PDB ID	XAA_GVDQ<br>hexamer<br>6NXM	XAA_GVDQ_calcium<br>trimer<br>6NY8
Resolution range	47.71 - 2.2 (2.279 - 2.2)	80.05 - 2.3 (2.382 - 2.3)
Space group	C 1 2 1	C 1 2 1
Unit cell	101.045 58.282 58.793 90 125.046 90	86.158 49.602 85.032 90 109.713 90
Total reflections	95755 (9491)	98944 (9361)
Unique reflections	14333 (1411)	15110 (1505)
Multiplicity	6.7 (6.7)	6.5 (6.2)
Completeness (%)	96.43 (89.65)	94.59 (85.71)
Mean I/sigma(I)	25.02 (2.53)	21.93 (1.90)
Wilson B-factor	50.43	55.74
R-merge	0.03291 (0.6889)	0.03765 (1.059)
R-meas	0.0358 (0.7469)	0.041 (1.156)
R-pim	0.01389 (0.2855)	0.01598 (0.457)
CC1/2	1 (0.905)	1 (0.864)
CC*	1 (0.975)	1 (0.963)
Reflections used in refinement	14333 (1265)	15110 (1308)
Reflections used for R-free	1202 (109)	1235 (111)
R-work	0.2457 (0.3578)	0.2875 (0.4163)
R-free	0.2770 (0.4159)	0.3041 (0.4535)
CC(work)	0.977 (0.806)	0.948 (0.858)
CC(free)	0.940 (0.681)	0.905 (0.842)
Number of non-hydrogen atoms	1874	1917
macromolecules	1868	1915
ligands		2
solvent	6	
Protein residues	274	272
RMS(bonds)	0.004	0.004
RMS(angles)	0.83	0.77
Ramachandran favored (%)	98.51	98.12
Ramachandran allowed (%)	1.49	1.88
Ramachandran outliers (%)	0	0
Rotamer outliers (%)	0.69	1.23
Clashscore	3.34	0
Average B-factor	72.91	79.41
macromolecules	72.93	79.41
ligands		80.45
solvent	68.55	

Table S4 | SAXS molecular weight measurements for select designs

Design	Expected trimer (MW)	SAXS condition	MW
XAX	33138	pH 7	34000
		pH 5	39000
XAA_GVDQ	32938	0 mM calcium	28000
		15 mM calcium	29000
		20 mM calcium	31000
		30 mM calcium	32000

Table S5 | NMR restraints and structural statistics for XAA structural ensembles
A set of structure calculation for XAA *de novo* using Chemical Shifts, NOEs and RDC data was performed.

Target	XAA de novo (PDB ID 6O0I)
**Distance constraints**	
Total NOE	92 [a]
Intraresidue	0
Inter-residue	
Sequential (|*i* – *j*| = 1)	0
Medium-range (|*i* – *j*| <= 4)	7
Long-range (|*i* – *j*| >= 5)	85
Intermolecular	66 [b]
Hydrogen bonds	0
Total dihedral angle restraints [c]	558
ϕ	279
ψ	279
**Structure statistics**	
RDC Q-factor (mean ± s.d.)	0.29 ± 0.03
Violations (mean ± s.d.)	
Distance constraints (Å)	
between 0 Å and 1 Å/structure	3.00 ± 2.65
between 1 Å and 2 Å/structure	1.50 ± 1.86
between 2 Å and 3 Å/structure	0.60 ± 1.80
above 3.0 Å/structure	0.00 ± 0.00
Deviations from idealized geometry	
Bond lengths (Å)	0.00 ± 0.00
Bond angles (°)	0.00 ± 0.00
Impropers (°)	0.00 ± 0.00
Average pairwise r.m.s. deviation (Å) [d]	
Heavy	1.00 ± 0.36
Backbone	1.22 ± 0.41

[a] These NOEs include 66 intramolecular restraints.

[b] 33 intermolecular NOE restraints are between chains A and B of the homo trimer. Remaining 33 restraints are duplicated between chains B and C to maintain symmetry within the system.

[c] Each molecule of the homo trimer utilizes 93 restraints. Dihedral angle restraints are utilized during fragment picking process. See Methods for more details.

[d] R.M.S. deviation was calculated over the structured region consisting of a-helices in the ensemble.

Table S6 | NMR restraints and structural statistics for k170 structural ensembles

Three sets of structure calculations for k170 (i) without calcium *de novo* using Chemical Shifts, NOEs and RDC data, (ii) without calcium using NMR Chemical Shifts and RDC data, and (iii) with calcium using NMR Chemical Shifts and RDC data were performed. k170 is a point mutant of XAA_GVDQ that removes a methionine from the plasmid backbone and behaves identically to XAA_GVDQ in NMR experiments (Fig. S15).

Target	k170 *de novo* without calcium (PDB ID 6O0C)	k170 without calcium	k170 with calcium
**Distance constraints**			
Total NOE	139 [a]	0	0
Intraresidue	0	0	0
Inter-residue			
Sequential ($\|i-j\|$ = 1)	0	0	0
Medium-range ($\|i-j\|$ <= 4)	23	0	0
Long-range ($\|i-j\|$ >= 5)	116	0	0
Intermolecular	56 [b]	0	0
Hydrogen bonds	0	0	0
Total dihedral angle restraints [c]	528	528	528
ϕ	264	264	264
ψ	264	264	264
**Structure statistics**			
RDC Q-factor (mean ± s.d.)	0.18 ± 0.01	0.21 ± 0.01	0.21 ± 0.01
Violations (mean ± s.d.)			
Distance constraints (Å)			
between 0 Å and 1 Å/structure	1.80 ± 1.80	0.00 ± 0.00	0.00 ± 0.00
between 1 Å and 2 Å/structure	0.30 ± 0.50	0.00 ± 0.00	0.00 ± 0.00
above 2.0 Å/structure	0.00 ± 0.00	0.00 ± 0.00	0.00 ± 0.00
Deviations from idealized geometry			
Bond lengths (Å)	0.00 ± 0.00	0.00 ± 0.00	0.00 ± 0.00
Bond angles (°)	0.00 ± 0.00	0.00 ± 0.00	0.00 ± 0.00
Impropers (°)	0.00 ± 0.00	0.00 ± 0.00	0.00 ± 0.00
Average pairwise r.m.s. deviation (Å) [d]			
Heavy	0.88 ± 0.20	0.52 ± 0.20	0.54 ± 0.21
Backbone	0.78 ± 0.17	0.47 ± 0.20	0.49 ± 0.23

[a] These NOEs include 83 intramolecular restraints.

[b] 28 intermolecular NOE restraints are between chains A and B of the homo trimer. Remaining 28 restraints are duplicated between chains B and C to maintain symmetry within the system.

[c] Each molecule of the homo trimer utilizes 88 restraints. Dihedral angle restraints are utilized during the fragment picking process. See Methods for more details.

[d] R.M.S. deviation was calculated over the structured region consisting of a-helices in the ensemble.

Supporting Text

Tuning of the free energy difference between short and long states

Crystal structures of designs XXA (PDB ID 6nyi) and XAX (PDB ID 6nye) match the modeled short state with Cα root-mean-square deviations (RMSDs) of 1.37 and 0.97, respectively at 1.9-2.3 Å resolution (Fig. 2a, b). For both, most of the deviation, as expected, is in the flexible hinge: the crystal structures show that each monomer adopts a different loop. For XXA, the crystal structure confirms the short state, but there are deviations from the model at the hydrogen bond network, which is not surprising since it is in a solvent exposed position. For XAX, the designed hydrogen bond network formed as expected (Fig 2b. "network A" panel vs. Fig. 2a "network A" panel). To further characterize the solution structure, we prepared a selective AI(LV)^{proS} methyl-labeled XAX sample. The 3D C_M-$C_M H_M$ NOESY spectrum for XAX shows NOE patterns consistent with the compact structure, with many unambiguously assigned close-range NOEs identified between the ^{13}Cδ1 methyl of Ile66 and ^{13}Cδ2 of Leu86 (Fig. 2c).

Attempts to solve a crystal structure for XAA were unsuccessful, but a ^{15}N MAI(LV)^{proS} methyl-labeled sample prepared for the protein showed highly dispersed NMR spectra. The fully assigned methyl HMQC spectra show resonances of increased linewidth, relative to our previously assigned XAX construct, indicating the presence of dynamics at the μsec-msec timescale. The fully assigned backbone amide TROSY-HSQC spectra confirm this result, and further reveal the presence of a minor component (~10-15% population, not exchanging with the major component at the NMR timescale, as confirmed by TROSY ZZ exchange experiments) for residues distributed at the interface between the inner and outer helices, (Fig. S14), likely due to local breaking of the regular C3 symmetry. Comparison of the shared methyl NOEs between the major state of XAA and XAA_GVDQ (which by NMR is locked in the short state) (Fig. S8a) and analysis of Residual Dipolar Couplings (RDCs) (Fig. S8b) further suggest that the flipping helix is in the short state, adopting an alternative packing against the inner helices. Finally, a comparison with the *de novo* NMR structure (solved using chemical shifts, long-range NOEs and RDCs; Table S5) clearly shows that the flipping helix is shifted by about a turn up towards the hinge (Fig. 2d,e). The C-terminal hydrogen bond network in XAA is solvent exposed and more likely to be disrupted by competing water interactions. This disrupted network likely destabilizes the interface between the flipping helix and the inner helices, allowing alternative packing. In contrast, the C-terminal region of XAX is hydrophobic, very well packed in the crystal structure and effectively stabilizes the flipping helix.

Design AAA (PDB ID 6nx2) adopts the long state according to crystallographic evidence with RMSD = 1.40 at about 2.3 Å resolution (Fig. 2e). While the helical propensity of the residues at and surrounding the hinge region was predicted to be low, in the crystal structure this region is clearly helical and the most flexible residues are at the C-terminus (Fig. S14). The structure of AAA deviates from the predicted model because the asparagines of two of the hydrogen bond networks coordinate bromide ions (ions and waters are not explicitly accounted for during modeling). In particular, N61 shifts toward the center of the helix (relative to the model to coordinate the ion (Fig. S10). Taken together, there is potential for a single sequence that can interconvert between the two designed states since a change in three residues, corresponding to the location of a hydrogen bond network, can switch the observed state (i.e., XAA vs AAA).

Supporting References

1. S. E. Boyken, *et al.*, De novo design of protein homo-oligomers with modular hydrogen-bond network-mediated specificity. *Science* **352**, 680–687 (2016).

2. M. V. Berjanskii, D. S. Wishart, A simple method to predict protein flexibility using secondary chemical shifts. *J. Am. Chem. Soc.* **127**, 14970–14971 (2005).

3. L. J. McGuffin, K. Bryson, D. T. Jones, The PSIPRED protein structure prediction server. *Bioinformatics* **16**, 404–405 (2000).
